# Supplementary material for: Improved visualization of high-dimensional data using the distance-of-distance transformation
Source: PLoS Comput Biol. 2022 Dec 20;18(12):e1010764. doi: 10.1371/journal.pcbi.1010764 (PMC9812310; doi:10.1371/journal.pcbi.1010764)
Supplement: S1 Text — (PDF) [file pcbi.1010764.s001.pdf]

Supporting information for: Improved visualization of high-dimensional data using the distance-of-distance transformation

Jinke Liu[1,2*], Martin Vinck[1,2]

1 Ernst Strüngmann Institute for Neuroscience in Cooperation with Max Planck Society, Frankfurt am Main, Germany
2 Donders Institute for Brain, Cognition and Behaviour, Nijmegen University, Nijmegen, Netherlands

* jinke.liu@esi-frankfurt.de

S1 Text. Influence of neighborhood size

In the following simulations, we will show that our method is relatively robust to the choice of neighborhood size K as long as K is smaller than the size of the clusters. However, as K approaches the sample number N, there could be distortions.

First, we generated 5 clusters following multivariate Gaussian distributions with different means. The size of each cluster was 20. The data contained another 200 noise points. The feature space had a dimensionality of 50. We found that with K ranging from 5 (smaller than the cluster size) to 20 (equal to the cluster size), the DoD transformation can robustly solve the scattering noise problem (S1A Fig). However, as K further increases, we can see that the t-SNE algorithm starts to introduce distortions in the low-dimensional embeddings. Moreover, in situations where there are more clusters, with K further approaching the total number of points N, the DoD transformation introduced significant distortions to the clusters and failed to put scattering noise points together (S1B Fig).

To explain this, consider two points from two distinct clusters. As K becomes very large, the sets of neighbors of these two points will start including more distant noise points. Because of this, the distance-of-distances between these cluster points will strongly shrink. Consequently, the advantage of the method is lost, because the shrinkage of the cluster and the noise points becomes comparable. Thus, it is generally desirable that K is not too large (ideally around or smaller than the cluster size).
