# Supplementary material for: Improved visualization of high-dimensional data using the distance-of-distance transformation
Source: PLoS Comput Biol. 2022 Dec 20;18(12):e1010764. doi: 10.1371/journal.pcbi.1010764 (PMC9812310; doi:10.1371/journal.pcbi.1010764)
Supplement: S2 Text — (PDF) [file pcbi.1010764.s002.pdf]

Supporting information for: Improved visualization of high-dimensional data using the distance-of-distance transformation

Jinke Liu[1,2*], Martin Vinck[1,2]

1 Ernst Strüngmann Institute for Neuroscience in Cooperation with Max Planck Society, Frankfurt am Main, Germany
2 Donders Institute for Brain, Cognition and Behaviour, Nijmegen University, Nijmegen, Netherlands

* jinke.liu@esi-frankfurt.de

S2 Text. Unsupervised noise detection

DoD transformation can be used as a noise detection method. This is based on the fact that after the DoD transformation, the noise point cloud no longer scatters, and its density increases. Therefore, we can use K-means or hierarchical clustering on the distance-of-distance matrix to identify the clusters. Then, we can compute how much the within-cluster distances change after the DoD transformation. Because the density changes more for noise clusters than data clusters, the noise cluster can be identified as the group of points that shows more within-cluster distance change.

Moreover, even without clustering, we can find scattering noise points simply by investigating how the set of neighbors changes for each point after the DoD transformation. The neighborhood of a noise point changes more compared to a non-noise point. Thus, the identity of neighbors changes more for noise points than for cluster points after the DoD transformation. In our simulation, we measure the changes in neighbor identities as the overlap rate of neighborhoods before and after the DoD transformation. A larger overlap rate indicates a smaller change. We find that the distribution of neighborhood overlap rate exhibit bi-modality. By using a midpoint method, we could calculate a threshold to bipartite the distribution. As an example, we simulated the unlabelled data points under the same experimental setting as in Figure 1. after the DoD transformation with $K = 5$, we found that the population with a smaller neighborhood overlap rate can be identified as noise points with high accuracy (S2 Fig).
