# Supplementary material for: Improved visualization of high-dimensional data using the distance-of-distance transformation
Source: PLoS Comput Biol. 2022 Dec 20;18(12):e1010764. doi: 10.1371/journal.pcbi.1010764 (PMC9812310; doi:10.1371/journal.pcbi.1010764)
Supplement: S3 Text — (PDF) [file pcbi.1010764.s003.pdf]

Supporting information for: Improved visualization of high-dimensional data using the distance-of-distance transformation

Jinke Liu[1,2*], Martin Vinck[1,2]

1 Ernst Strüngmann Institute for Neuroscience in Cooperation with Max Planck Society, Frankfurt am Main, Germany
2 Donders Institute for Brain, Cognition and Behaviour, Nijmegen University, Nijmegen, Netherlands

* jinke.liu@esi-frankfurt.de

S3 Text. PCA preprocessing

PCA as a dimensionality reduction method is often used in combination with t-SNE, mainly to improve computational efficiency by reducing the dimensionality of the data set before running t-SNE [1]. There are two ways to incorporate PCA into the t-SNE analysis. First of all, PCA can be used to initialize t-SNE embeddings. For example, we can use the first two principal components as the initialized 2D representations of the data set, and then t-SNE can carry out gradient descent steps from such initialized coordinates. Secondly, PCA can be used as a preprocessing step to reduce the dimensionality of the original data set. This is a standard technique to make the computation more efficient [1]. By keeping only the first few principal components, we could capture most of the variability in our data with reduced dimensionality.

In our experiments, we found that both PCA initialization and PCA preprocessing did not resolve the scattering noise problem (S3 Fig). Furthermore, PCA can only be applied to data sets, but not directly to distance matrices. In situations where dissimilarity was calculated not simply as Manhattan / Euclidean distance (e.g. distances among correlation matrices, or optimal transport distances over spiking patterns [2]), it is no longer feasible to apply PCA as a preprocessing step. Overall, the DoD transformation is another useful technique that can be used together with other techniques like PCA. In fact, both methods can be combined with their own advantages.

References

1. Kobak D, Berens P. The art of using t-SNE for single-cell transcriptomics. Nature communications. 2019;10(1):1–14.

2. Grossberger L, Battaglia FP, Vinck M. Unsupervised clustering of temporal patterns in high-dimensional neuronal ensembles using a novel dissimilarity measure. PLoS computational biology. 2018;14(7):e1006283.
