# Supplementary material for: Improved visualization of high-dimensional data using the distance-of-distance transformation
Source: PLoS Comput Biol. 2022 Dec 20;18(12):e1010764. doi: 10.1371/journal.pcbi.1010764 (PMC9812310; doi:10.1371/journal.pcbi.1010764)
Supplement: S4 Text — (PDF) [file pcbi.1010764.s004.pdf]

Supporting information for: Improved visualization of high-dimensional data using the distance-of-distance transformation

Jinke Liu[1,2*], Martin Vinck[1,2]

1 Ernst Strüngmann Institute for Neuroscience in Cooperation with Max Planck Society, Frankfurt am Main, Germany
2 Donders Institute for Brain, Cognition and Behaviour, Nijmegen University, Nijmegen, Netherlands

* jinke.liu@esi-frankfurt.de

S4 Text. Fewer noise points than cluster points

In situations where the number of noise points is smaller than the number of non-noise points, DoD transformation still succeeded in putting all noise points together as a separate cluster (S4 Fig).
