# Supplementary material for: Improved visualization of high-dimensional data using the distance-of-distance transformation
Source: PLoS Comput Biol. 2022 Dec 20;18(12):e1010764. doi: 10.1371/journal.pcbi.1010764 (PMC9812310; doi:10.1371/journal.pcbi.1010764)
Supplement: S5 Text — (PDF) [file pcbi.1010764.s005.pdf]

Supporting information for: Improved visualization of high-dimensional data using the distance-of-distance transformation

Jinke Liu[1,2*], Martin Vinck[1,2]

1 Ernst Strüngmann Institute for Neuroscience in Cooperation with Max Planck Society, Frankfurt am Main, Germany
2 Donders Institute for Brain, Cognition and Behaviour, Nijmegen University, Nijmegen, Netherlands

* jinke.liu@esi-frankfurt.de

S5 Text. Influence of perplexity

Perplexity is an important tunable parameter for the t-SNE algorithm. In the original t-SNE paper, the author suggested that the choice of perplexity value within a reasonable range between 5 and 50 did not seem to influence the experiment results [1]. However, in practice, it has been suggested to use 1% of the total sample size of the data set [2]. Different choices of perplexity change the behavior of t-SNE [3]. To test whether the scattering noise problem can be solved by the t-SNE algorithm itself, we carefully tuned the perplexity value based on the number of points in each cluster and the number of clusters. However, we did not observe the alleviation of the "scattering noise problem". With a higher perplexity value, some local geometrical structures may emerge in the Gaussian clusters as they became more ellipsoidal and also in the random noise cloud. Overall, we observed that there existed no perplexity value that could make noise points attracted to each other, and the scattering noise problem remained (S5 Fig).

References

1. Maaten Lvd, Hinton G. Visualizing data using t-SNE. Journal of machine learning research. 2008;9(Nov):2579–2605.

2. Kobak D, Berens P. The art of using t-SNE for single-cell transcriptomics. Nature communications. 2019;10(1):1–14.

3. Wattenberg M, Viégas F, Johnson I. How to use t-SNE effectively. Distill. 2016;1(10):e2.
