# Supplementary material for: Improved visualization of high-dimensional data using the distance-of-distance transformation
Source: PLoS Comput Biol. 2022 Dec 20;18(12):e1010764. doi: 10.1371/journal.pcbi.1010764 (PMC9812310; doi:10.1371/journal.pcbi.1010764)
Supplement: S6 Text — (PDF) [file pcbi.1010764.s006.pdf]

Supporting information for: Improved visualization of high-dimensional data using the distance-of-distance transformation

Jinke Liu[1,2*], Martin Vinck[1,2]

1 Ernst Strüngmann Institute for Neuroscience in Cooperation with Max Planck Society, Frankfurt am Main, Germany
2 Donders Institute for Brain, Cognition and Behaviour, Nijmegen University, Nijmegen, Netherlands

* jinke.liu@esi-frankfurt.de

S6 Text. Distortion in noise-free situations

Next, we quantify the downsides of applying DoD transformation in the absence of scattering noise points in the data set. Firstly, we applied the DoD transformation to a simulated noise-free data set and examined whether the relative positions across the cluster centers were maintained. We found that the separation of clusters is maintained for a small K. For K larger than the cluster size, though we observed some distortions in the shape of clusters, the separation between clusters was largely preserved, as it is indicated by the high correlation coefficient between dissimilarity matrices calculated from the low-dimensional embeddings (S6A Fig). Similarly, we generated a high-dimensional noise-free data set where one of the clusters had a relatively lower density (S6B Fig). We found that the separation of clusters was improved for both small and large K. However, the exact location of the clusters shows variability, which is due to t-SNE itself based on the random seed used for initialization [1].

References

1. Kobak D, Berens P. The art of using t-SNE for single-cell transcriptomics. Nature communications. 2019;10(1):1–14.
