# Supplementary material for: Improved visualization of high-dimensional data using the distance-of-distance transformation
Source: PLoS Comput Biol. 2022 Dec 20;18(12):e1010764. doi: 10.1371/journal.pcbi.1010764 (PMC9812310; doi:10.1371/journal.pcbi.1010764)
Supplement: S7 Text — (PDF) [file pcbi.1010764.s007.pdf]

Supporting information for: Improved visualization of high-dimensional data using the distance-of-distance transformation

Jinke Liu[1,2*], Martin Vinck[1,2]

1 Ernst Strüngmann Institute for Neuroscience in Cooperation with Max Planck Society, Frankfurt am Main, Germany
2 Donders Institute for Brain, Cognition and Behaviour, Nijmegen University, Nijmegen, Netherlands

* jinke.liu@esi-frankfurt.de

S7 Text. Allen Institute Brain observatory electrophysiological recordings

Data were downloaded in NWB format from the Allen Neuropixel Brain Observatory [1]. There are in total 32 adult mice in the brain observatory 1.1 paradigm with an average age of 112 days. Among them 27 are male and 5 are female. 16 of them are wild-type mice, and the rest of them are transgenic lines (N = 6, Sst-IRES-Cre/wt; N = 5, Pvalb-IRES-Cre/wt; N = 5, Vip-IRES-Cre/wt). Rodents were head-fixed and presented with different types of visual stimuli in a certain order. In total, six high-throughput Neuropixel probes were inserted into the mouse brain and recorded simultaneously from both visual cortical areas and subcortical areas including hippocampus and thalamus. For visual neurons, we included only the units with a signal-to-noise ratio larger than 0.5. We estimated the direction selectivity of a single neuron by its average response to drifting grating stimuli. By investigating the tuning curves of the recorded visual neurons, we found that the majority of them had two peaks with a difference of 180 degrees. The bimodality of the tuning curve suggested that most neurons are orientation-selective instead of direction-selective (S7B Fig).

References

1. Siegle JH, Jia X, Durand S, Gale S, Bennett C, Graddis N, et al. A survey of spiking activity reveals a functional hierarchy of mouse corticothalamic visual areas. Biorxiv. 2019; p. 805010.
