# Supplementary material for: Improved visualization of high-dimensional data using the distance-of-distance transformation
Source: PLoS Comput Biol. 2022 Dec 20;18(12):e1010764. doi: 10.1371/journal.pcbi.1010764 (PMC9812310; doi:10.1371/journal.pcbi.1010764)
Supplement: S8 Text — (PDF) [file pcbi.1010764.s008.pdf]

Supporting information for: Improved visualization of high-dimensional data using the distance-of-distance transformation

Jinke Liu[1,2*], Martin Vinck[1,2]

1 Ernst Strüngmann Institute for Neuroscience in Cooperation with Max Planck Society, Frankfurt am Main, Germany
2 Donders Institute for Brain, Cognition and Behaviour, Nijmegen University, Nijmegen, Netherlands

* jinke.liu@esi-frankfurt.de

S8 Text. Improvement of classification

We show that KNN classification accuracy on the stimulus-evoked responses was higher for distance-of-distance matrices even without t-SNE (S8 Fig). Here, we trained the KNN on both original high-dimensional data and t-SNE embeddings with the presence of noise. In both cases, the average cross-validated accuracy on the test data set was higher.
