# Supplementary material for: Improved visualization of high-dimensional data using the distance-of-distance transformation
Source: PLoS Comput Biol. 2022 Dec 20;18(12):e1010764. doi: 10.1371/journal.pcbi.1010764 (PMC9812310; doi:10.1371/journal.pcbi.1010764)
Supplement: S9 Text — (PDF) [file pcbi.1010764.s009.pdf]

Supporting information for: Improved visualization of high-dimensional data using the distance-of-distance transformation

Jinke Liu[1,2*], Martin Vinck[1,2]

1 Ernst Strüngmann Institute for Neuroscience in Cooperation with Max Planck Society, Frankfurt am Main, Germany
2 Donders Institute for Brain, Cognition and Behaviour, Nijmegen University, Nijmegen, Netherlands

* jinke.liu@esi-frankfurt.de

S9 Text. Distortion of real neural data by DoD transformation

To investigate whether the DoD transformation may distort the continuous structure in data in the absence of noise, we analyzed a calcium imaging data set from mouse V1 [1]. Mice were shown a visual grating stimulus that moves in 32 different directions. By visualizing the neural activities in a 2D embedding, it can be observed that the trials are organized in a continuous ring structure. This is expected as stimulus direction is a circular variable. After applying the DoD transformation to the distance matrix, we can see that the ring structure was largely maintained (S9 Fig). The distance matrices had similar structures and were highly correlated ($\rho = 0.875$). In addition, we trained a KNN classifier on both the original neural representations and on the 2D embeddings. We found that after the DoD transformation, the cross-validated performance decreased only from 94.3% to 91.8% on high-dimensional neural representations, and it decreased only from 88.7% to 87.4% on the low-dimensional embeddings. This suggests that DoD transformation introduces very limited distortions and preserves the geometry in the original manifold, even for a continuous structure.

Next, we applied the transformation to neural spiking data as in Figure 5 but without spontaneous activities. We found that the separation between clusters corresponding to different stimulus orientations increased with the DoD transformation (S10 Fig). This result suggests that DoD transformation can improve the clustering even for a noise-free data set.

References

1. Stringer C, Pachitariu M, Steinmetz N, Carandini M, Harris KD. High-dimensional geometry of population responses in visual cortex. Nature. 2019; p. 1.
