# Supplementary material for: Improved visualization of high-dimensional data using the distance-of-distance transformation
Source: PLoS Comput Biol. 2022 Dec 20;18(12):e1010764. doi: 10.1371/journal.pcbi.1010764 (PMC9812310; doi:10.1371/journal.pcbi.1010764)
Supplement: S10 Text — (PDF) [file pcbi.1010764.s010.pdf]

Supporting information for: Improved visualization of high-dimensional data using the distance-of-distance transformation

Jinke Liu[1,2*], Martin Vinck[1,2]

1 Ernst Strüngmann Institute for Neuroscience in Cooperation with Max Planck Society, Frankfurt am Main, Germany
2 Donders Institute for Brain, Cognition and Behaviour, Nijmegen University, Nijmegen, Netherlands

* jinke.liu@esi-frankfurt.de

S10 Text. Distortion of real convolutional neural network data by DoD transformation

To further test whether DoD transformation can alter the geometry of noise-free real data, we applied the transformation to convolutional neural network representations of image patches. We found that with a modest neighborhood size ($K = 5$), the relationships between clusters of different objects were largely maintained. We found that after the DoD transformation, KNN classification performance increased slightly from 98.8% to 98.9% on high-dimensional neural representations, and it decreased slightly from 98.7% to 98.5% on the low-dimensional embeddings (S11 Fig).
