# Supplementary material for: Improved visualization of high-dimensional data using the distance-of-distance transformation
Source: PLoS Comput Biol. 2022 Dec 20;18(12):e1010764. doi: 10.1371/journal.pcbi.1010764 (PMC9812310; doi:10.1371/journal.pcbi.1010764)
Supplement: S11 Text — (PDF) [file pcbi.1010764.s011.pdf]

Supporting information for: Improved visualization of high-dimensional data using the distance-of-distance transformation

Jinke Liu[1,2*], Martin Vinck[1,2]

1 Ernst Strüngmann Institute for Neuroscience in Cooperation with Max Planck Society, Frankfurt am Main, Germany
2 Donders Institute for Brain, Cognition and Behaviour, Nijmegen University, Nijmegen, Netherlands

* jinke.liu@esi-frankfurt.de

S11 Text. Application of DoD transformation to CNN representation of images from different data sets

We then analyzed t-SNE embeddings for sketch image patches that contain randomly 5 out of 20 different object classes. The t-SNE embeddings showed a clear clustering for different object classes (S12A Fig). Then we randomly selected images from ImageNet data set. We observed that the embedding coordinates for these ImageNet patches overlapped with the sketch image patches (S12B Fig). At first sight, this seems to suggest that the ImageNet images lie in the same representational space as the sketch image patches, and that their VGG16 representations exhibit a high degree of similarity to the sketch objects. However, it is likely that the ImageNet image patches would have caused a scattering noise problem because they have large distances to all the other image patches, both the sketch and the ImageNet ones. Because VGG16 representations live in a high-dimensional space, we predicted that the DoD transformation should lead to a separation of the ImageNet image patches from the sketch image patches. Indeed, the DoD transformation relocated the activation patterns of ImageNet image patches into a separate region of the low-dimensional embedding, while preserving the geometry of object relationships as compared to the original t-SNE without ImageNet image patches (S12C Fig). This shows that the VGG16 representations of sketch image patches indeed lie in a different region of the low-dimensional embedding space than the representations of ImageNet image patches. Thus, we further confirmed that the DoD transformation is generally a useful technique to help visualize the representation of images by convolutional neural networks.
