# Supplementary material for: Improved visualization of high-dimensional data using the distance-of-distance transformation
Source: PLoS Comput Biol. 2022 Dec 20;18(12):e1010764. doi: 10.1371/journal.pcbi.1010764 (PMC9812310; doi:10.1371/journal.pcbi.1010764)
Supplement: S12 Text — (PDF) [file pcbi.1010764.s012.pdf]

Supporting information for: Improved visualization of high-dimensional data using the distance-of-distance transformation

Jinke Liu[1,2*], Martin Vinck[1,2]

1 Ernst Strüngmann Institute for Neuroscience in Cooperation with Max Planck Society, Frankfurt am Main, Germany
2 Donders Institute for Brain, Cognition and Behaviour, Nijmegen University, Nijmegen, Netherlands

* jinke.liu@esi-frankfurt.de

S12 Text. Runtime of DoD transformation

We can see in S13 Fig that the run time of DoD transformation increases exponentially with the number of data points N. A larger neighborhood size K could also increase the run time a bit, especially when the distance matrix is large. And as it is shown in Figure 3C/D as the number of data points N increases, the effectiveness of DoD transformation decreases. With a fixed dimensionality D, larger the data set size, less effective the DoD transformation and larger the time cost. These disadvantages can only be compensated if the data points are living in a higher dimension.
