# Supplementary figures and images for: Improved visualization of high-dimensional data using the distance-of-distance transformation
Source: PLoS Comput Biol. 2022 Dec 20;18(12):e1010764. doi: 10.1371/journal.pcbi.1010764 (PMC9812310; doi:10.1371/journal.pcbi.1010764)

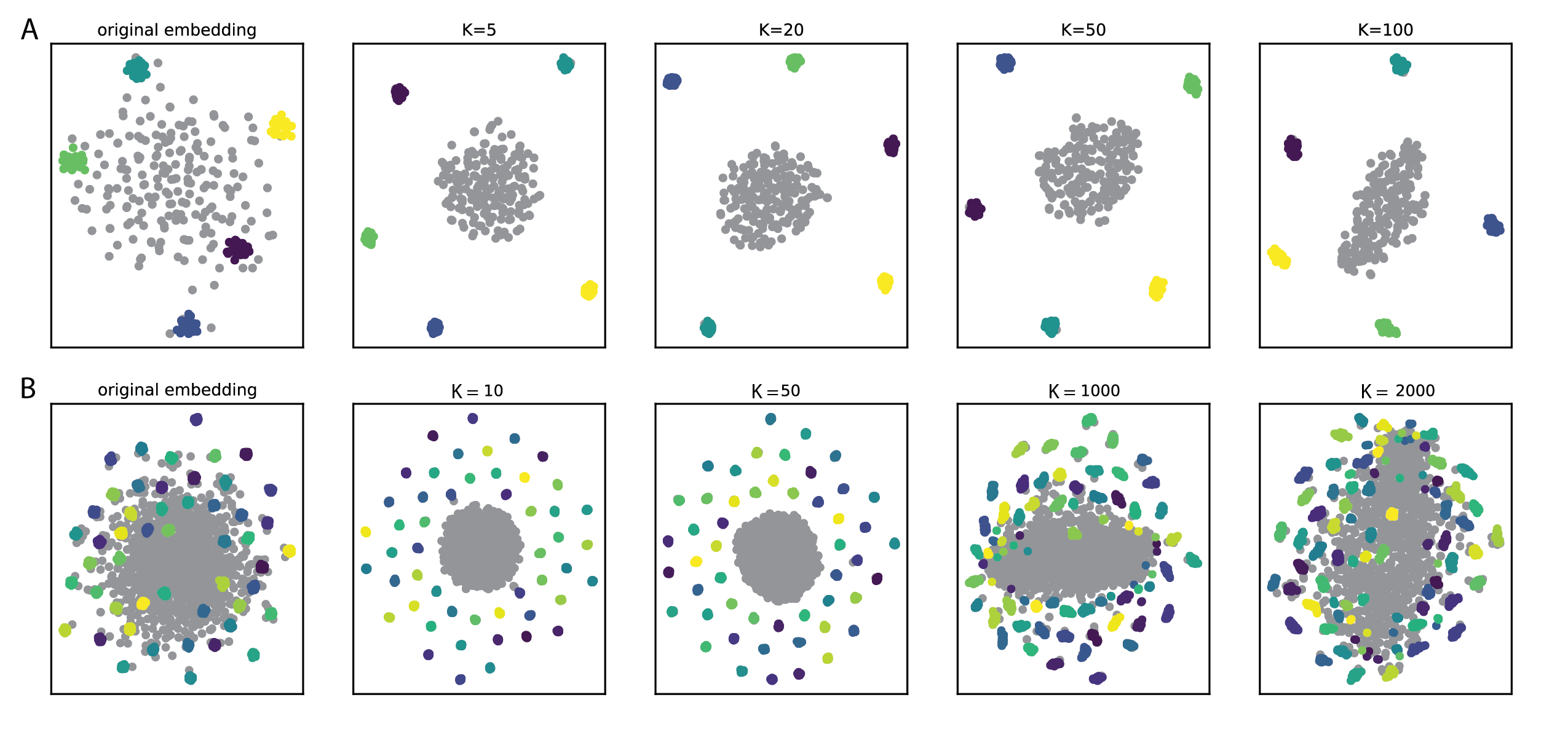

Supplement: S1 Fig — A: 5 clusters, each with 20 points; 200 scattering noise points; dimensionality of 50; Original embedding (left) and DoD transformation with a neighborhood size of 5, 20, 50 and 100. B: 50 clusters, each with 20 points; 1000 scattering noise points; dimensionality of 50; Original embedding (left) and DoD transformation with a neighborhood size of 10, 50, 1000 and 2000. (PNG) [file pcbi.1010764.s013.png]

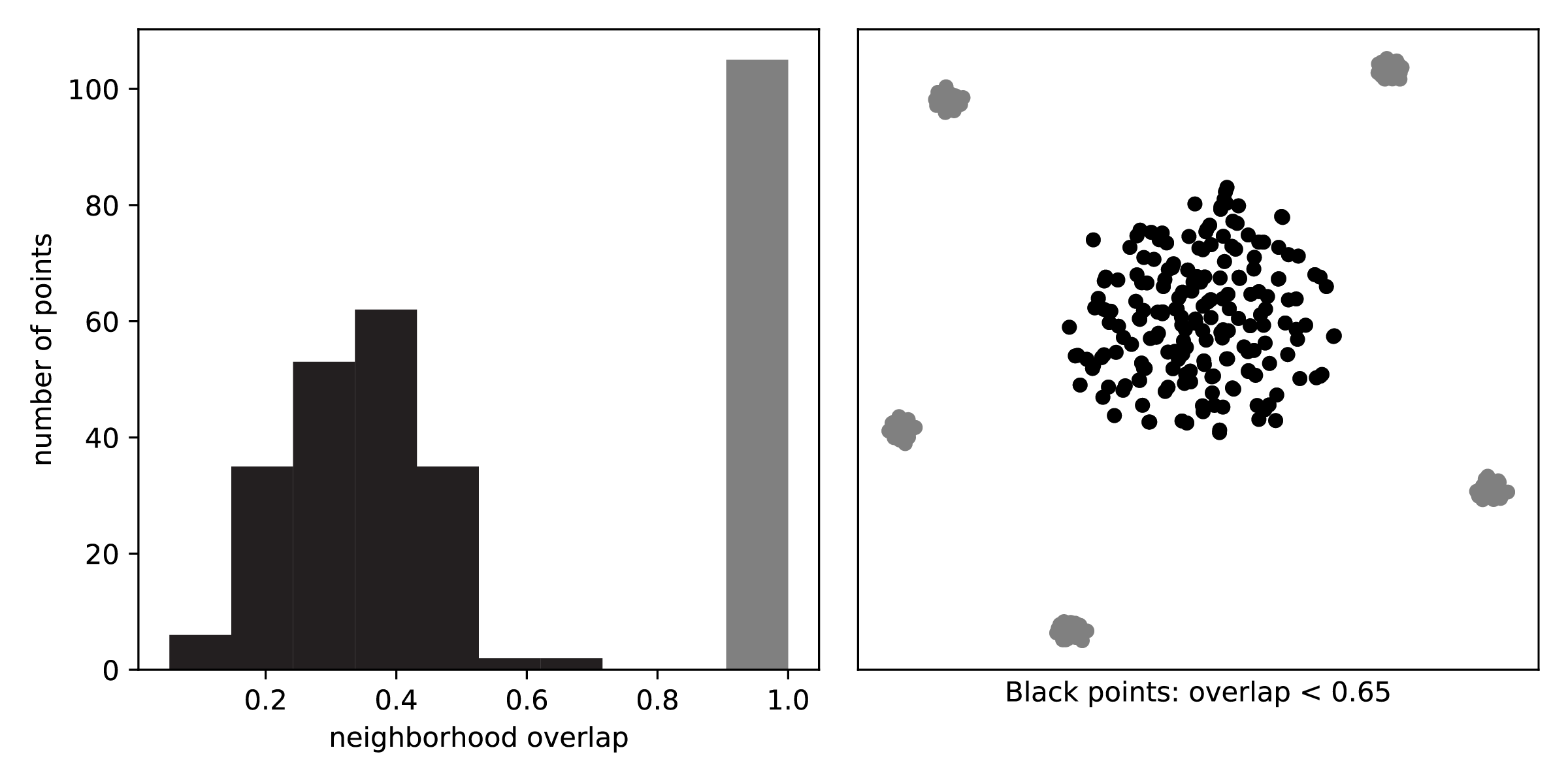

Supplement: S2 Fig — A: Distribution of overlap rate of neighborhood identity before and after the DoD transformation. B: Points with smaller overlap rate (< 65%) were identified as scattering noise points (black). (PNG) [file pcbi.1010764.s014.png]

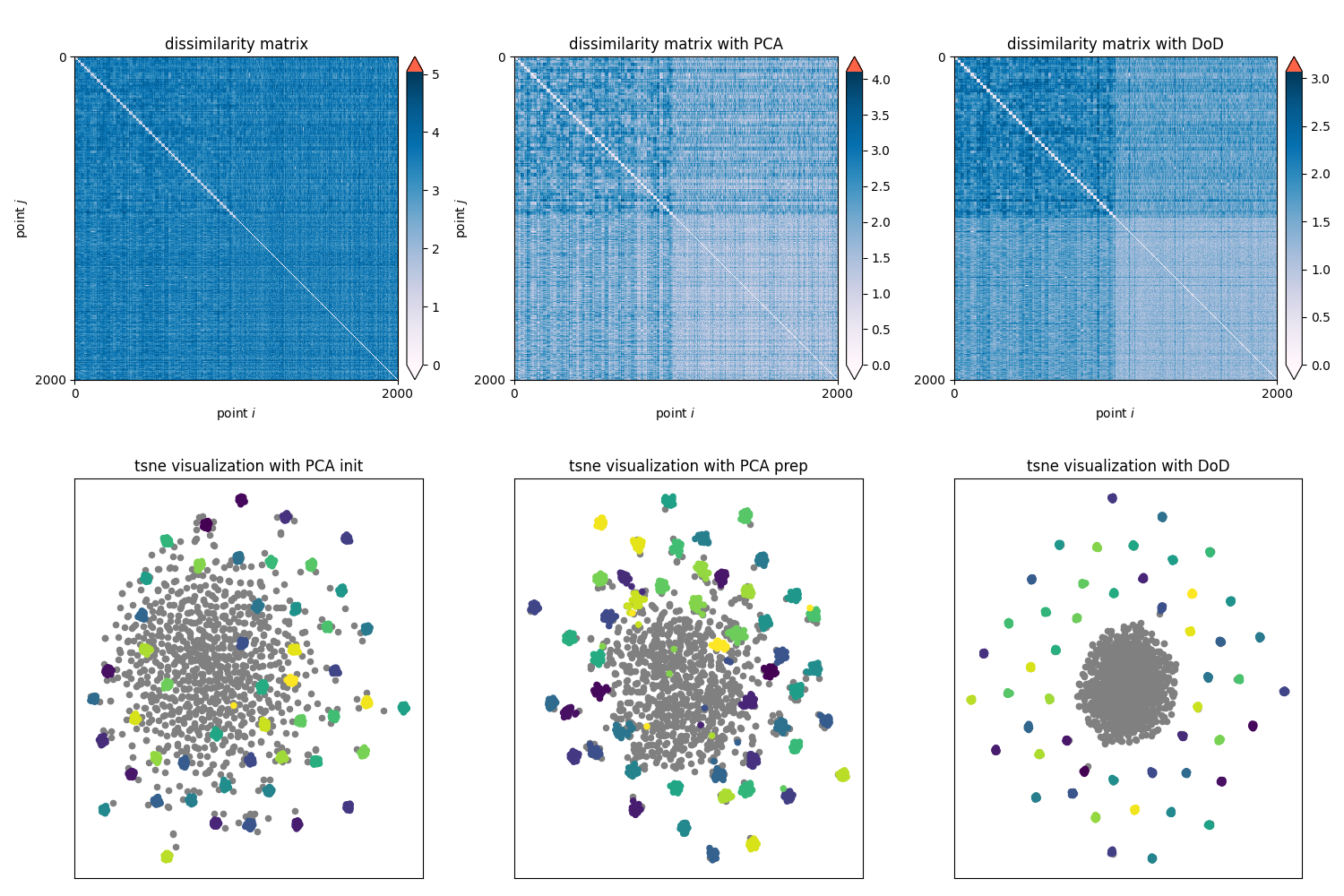

Supplement: S3 Fig — 50 clusters, each with 20 points; 1000 scattering noise points; dimensionality of 50; PCA preprocessing uses the first 10 principal components; DoD transformation uses neighborhood size of 10. (PNG) [file pcbi.1010764.s015.png]

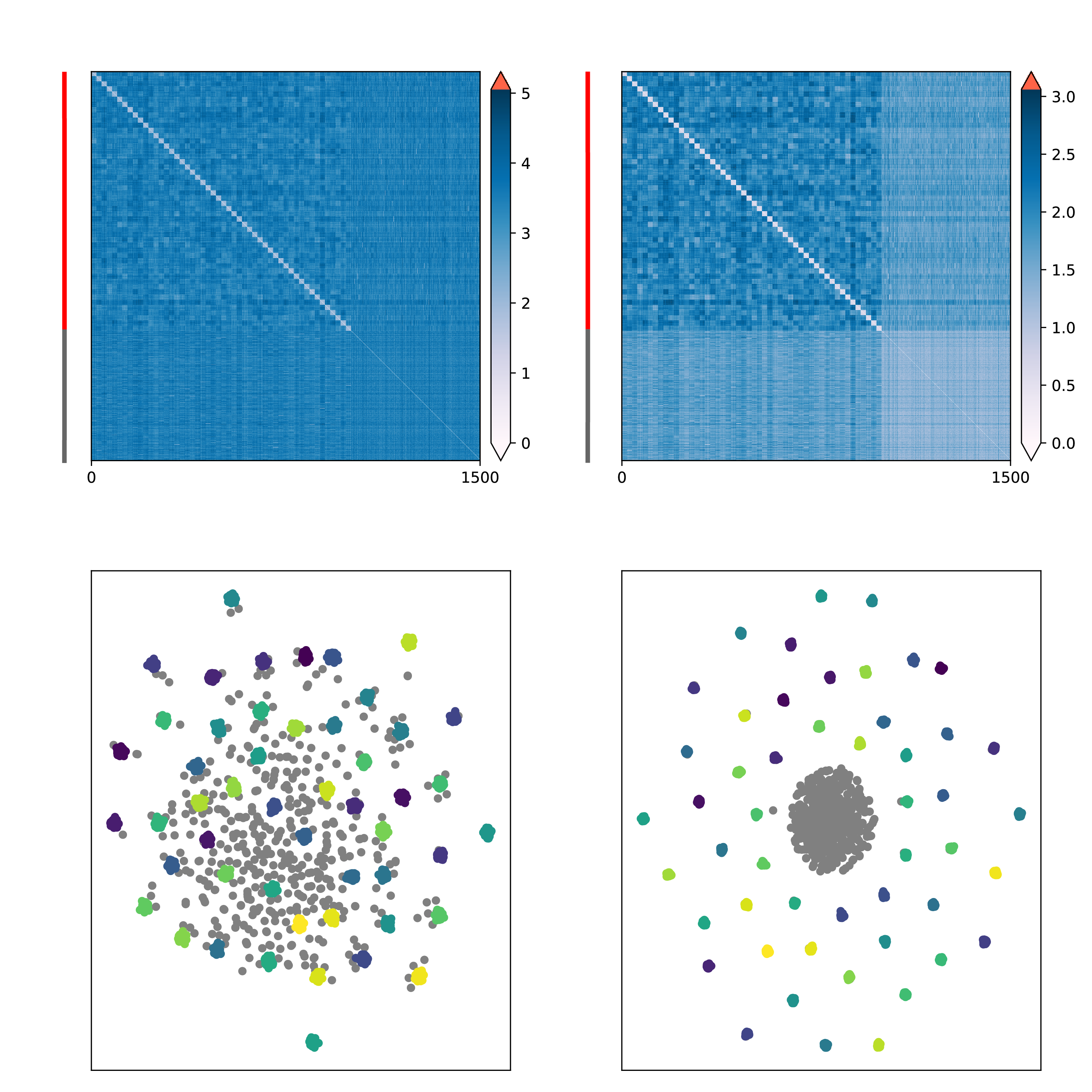

Supplement: S4 Fig — 50 clusters, each with 20 points; 500 noise points; dimensionality of 50; DoD transformation with a neighborhood size of 5. Left, original t-SNE visualization. Right, t-SNE visualization with DoD transformation. (PNG) [file pcbi.1010764.s016.png]

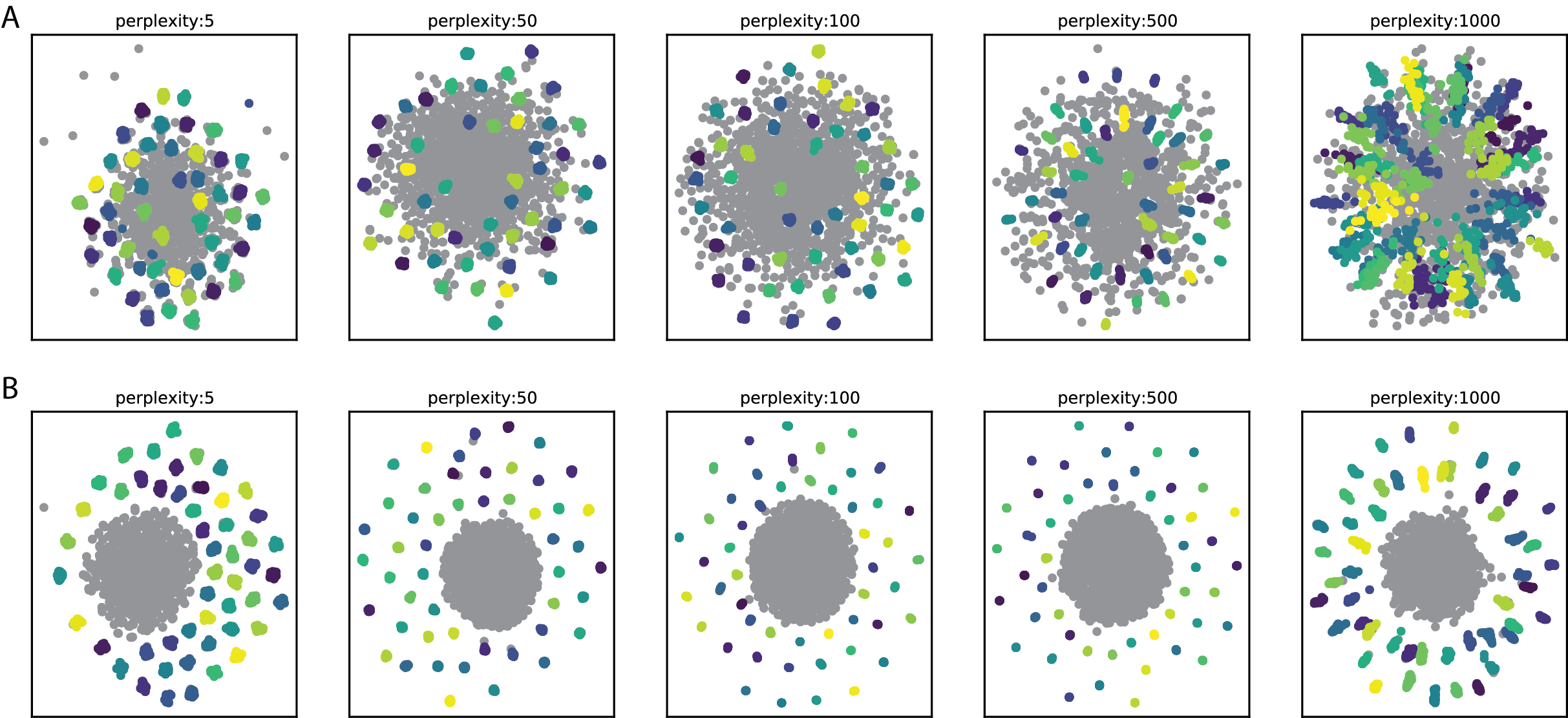

Supplement: S5 Fig — 50 clusters, each with 20 points; 1000 noise points; dimensionality of 50; DoD transformation with a neighborhood size of 5; perplexity values are 5, 50, 100, 500, 1000 from left to right. A: t-SNE on original distance matrix. B: t-SNE on distance matrix after DoD transformation. (PNG) [file pcbi.1010764.s017.png]

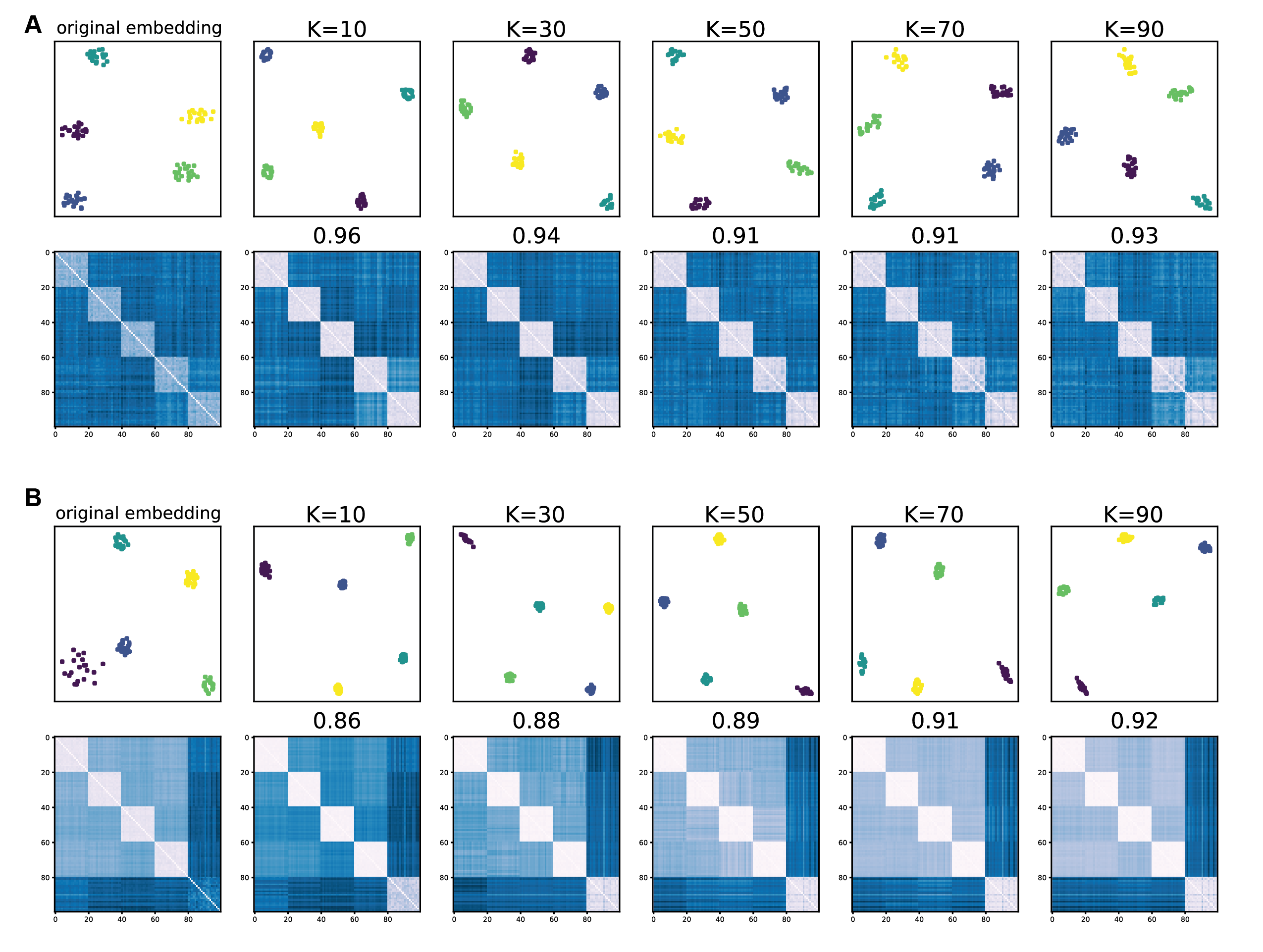

Supplement: S6 Fig — A: 5 clusters, each with 20 points; dimensionality of 50; DoD transformation with neighborhood size ranging from 5 to 30. B: 5 clusters, each with 20 points; dimensionality of 50; DoD transformation with neighborhood size ranging from 5 to 30. All clusters were generated from multivariate Gaussian distribution and one of them is with a larger standard deviation (0.5) than the rest (0.2). (PNG) [file pcbi.1010764.s018.png]

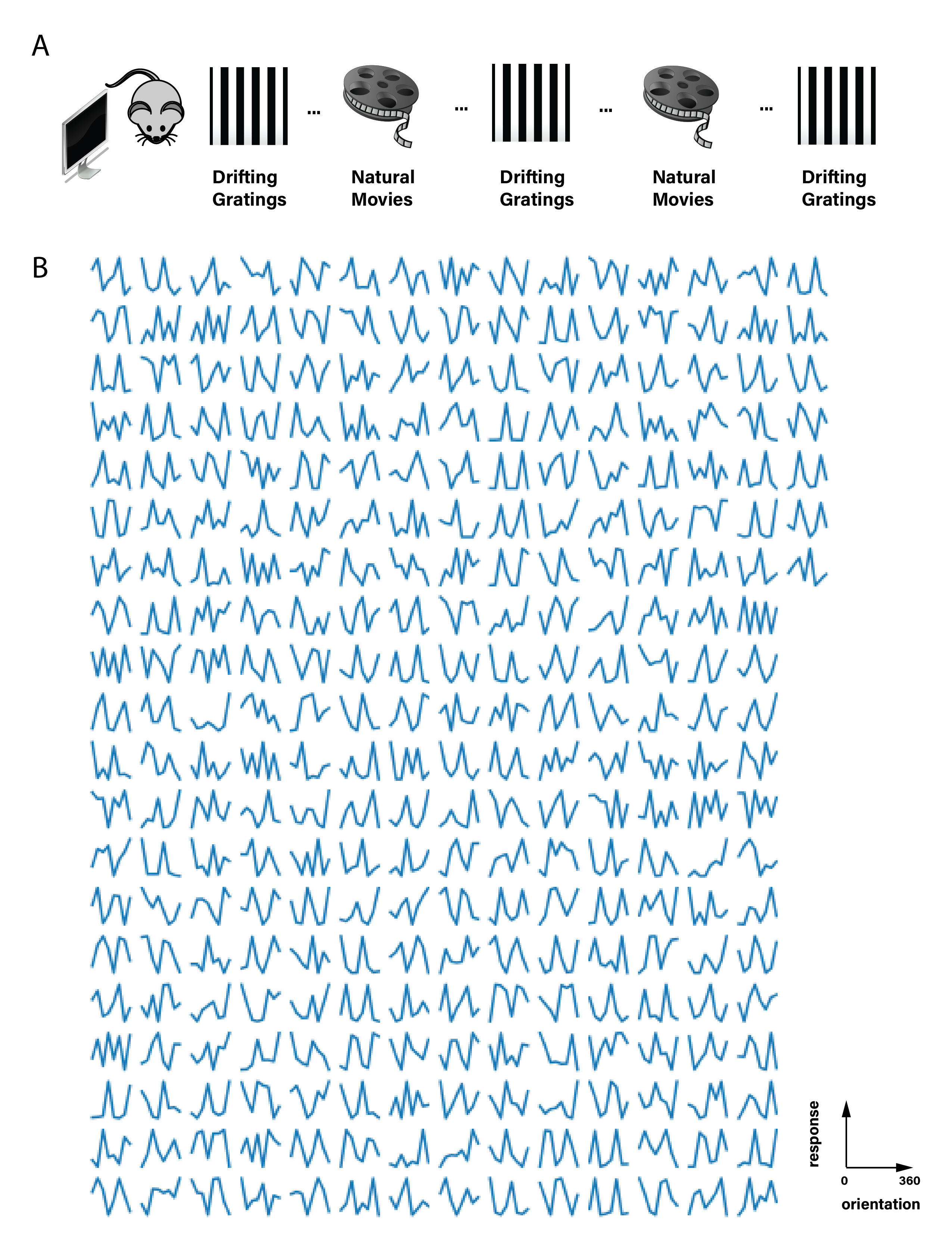

Supplement: S7 Fig — A: Illustration of recording session of drifting grating visual stimulus. B: Direction tuning curves of recorded units in session 754829445. (PNG) [file pcbi.1010764.s019.png]

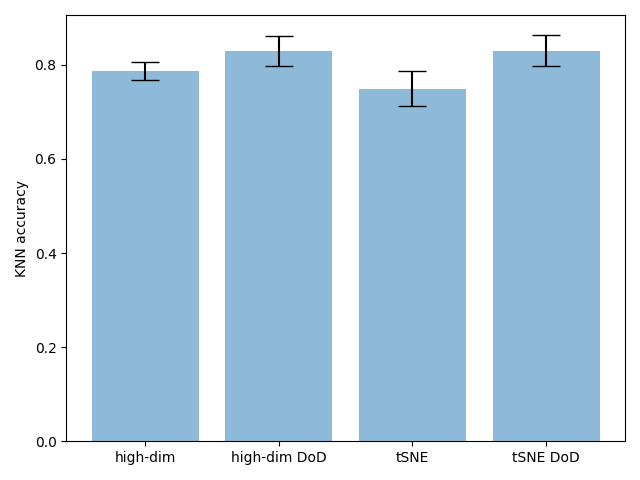

Supplement: S8 Fig — For both the distances in the high-dimensional space and the distances in the low-dimensional embedding, the cross-validated KNN classification scores are higher after DoD transformation. (PNG) [file pcbi.1010764.s020.png]

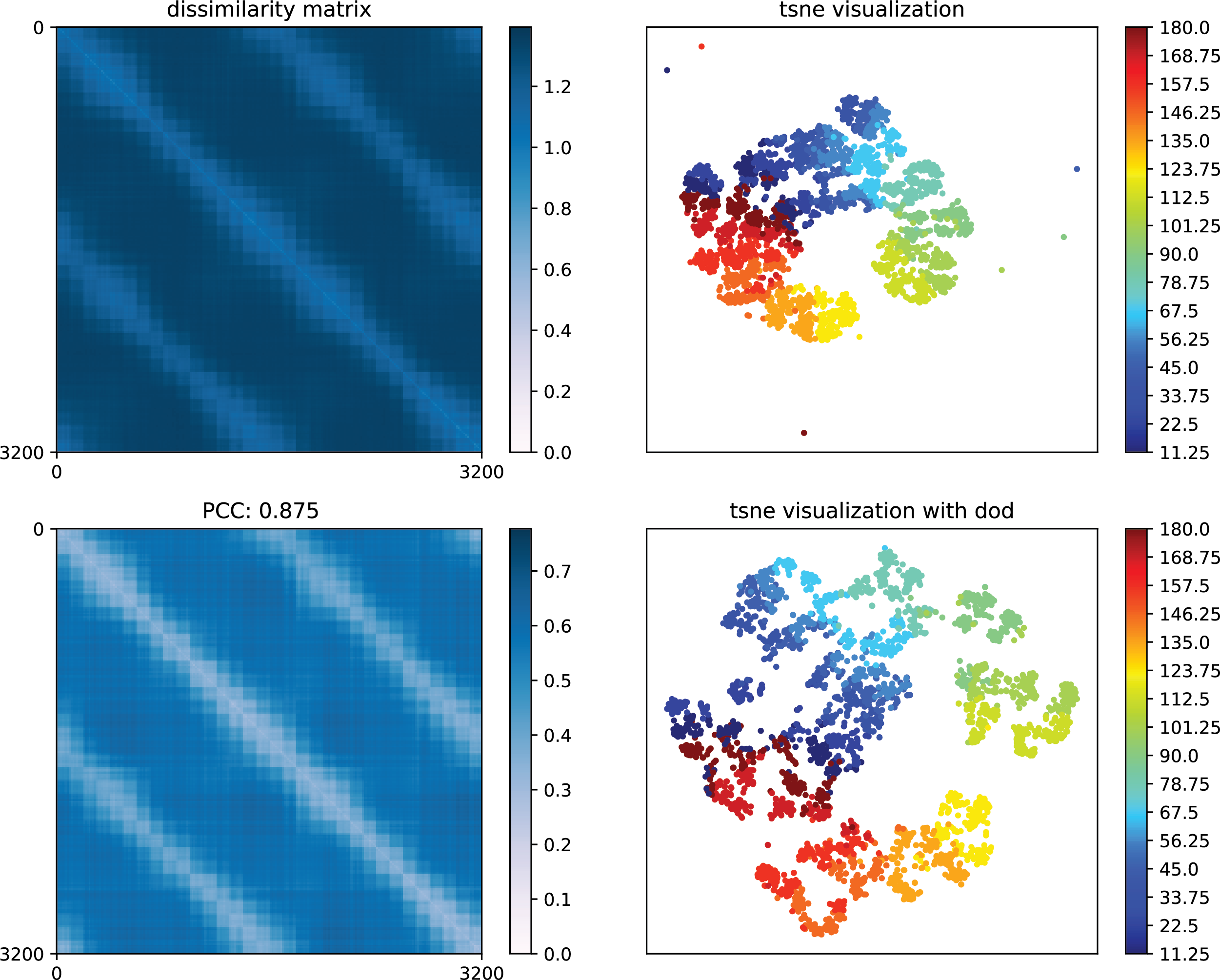

Supplement: S9 Fig — Each data point represents the populational firing pattern in a given trial (n = 3200). Trials with different stimulus orientations are labelled in different colors. (PNG) [file pcbi.1010764.s021.png]

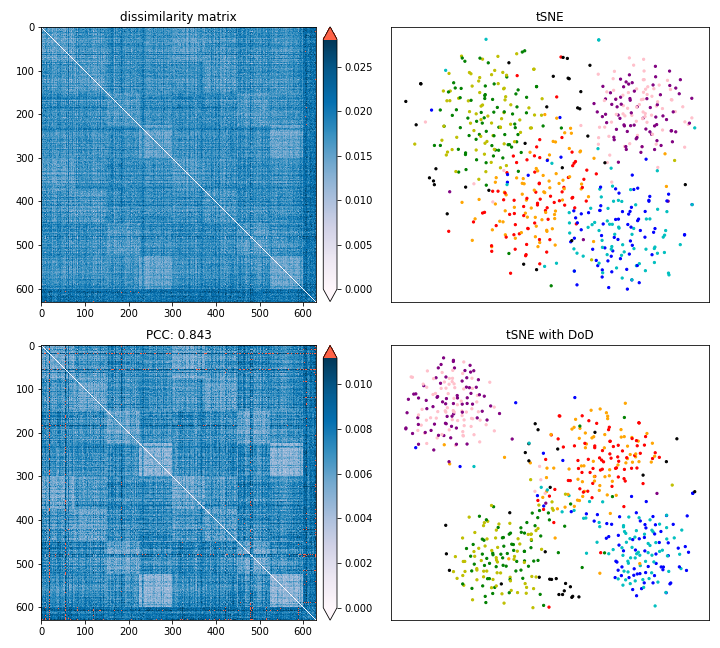

Supplement: S10 Fig — Each data point represents the populational spiking pattern in a given trial (n = 600). Trials with different stimulus orientations are labelled in different colors. (PNG) [file pcbi.1010764.s022.png]

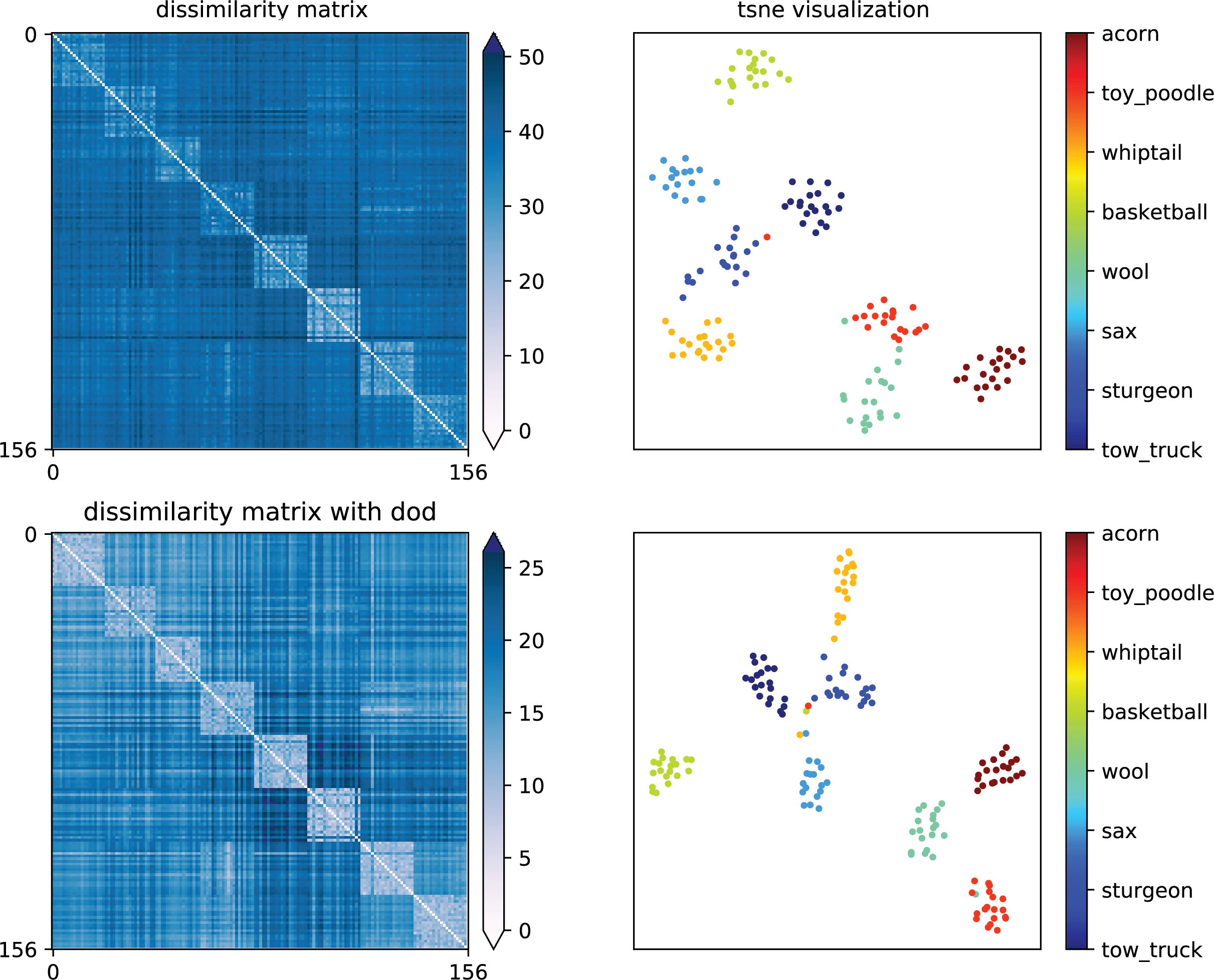

Supplement: S11 Fig — 2D t-SNE embeddings for 8 random ImageNet classes maintain clustering patterns after the DoD transformation. (PNG) [file pcbi.1010764.s023.png]

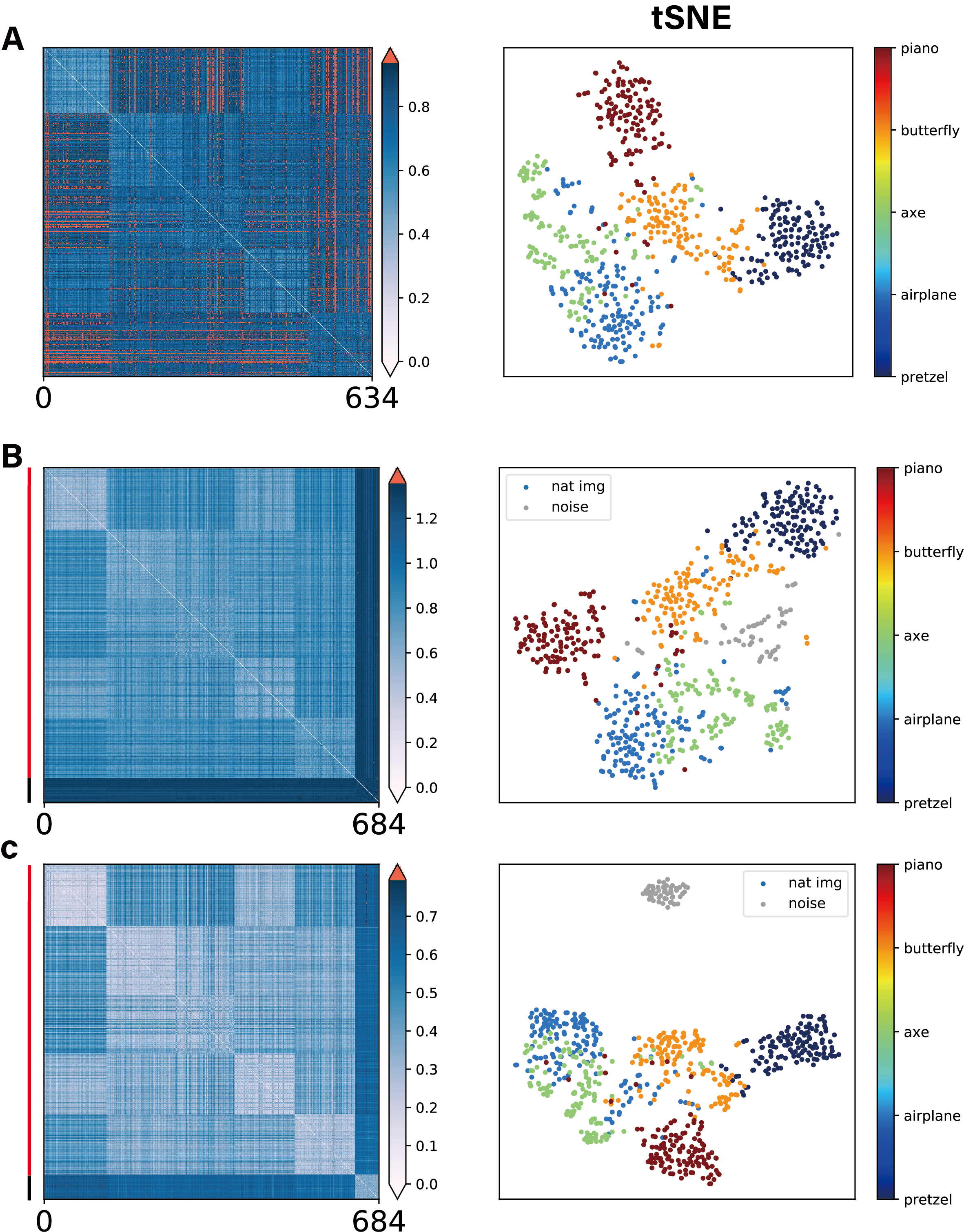

Supplement: S12 Fig — A: Low-dimensional manifold for 634 sketch patches from 5 different classes. Distances were computed based on the high-dimensional vectors in the fully connected layer of the pretrained Alexnet network. B: Low-dimensional manifold for data including both sketch patches and 50 random ImageNet patches. C: DoD transformation separates ImageNet patches from sketch patches. Left, Euclidean distance matrix. Right, 2D t-SNE embeddings. Sketch images of different classes are labelled in different colors. ImageNet patches are labelled in grey. (PNG) [file pcbi.1010764.s024.png]

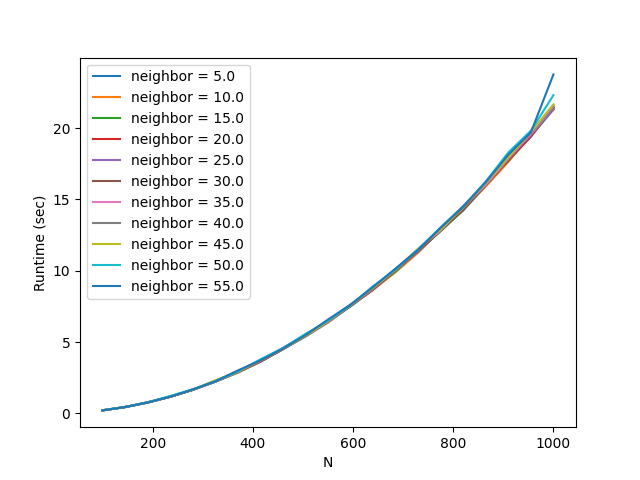

Supplement: S13 Fig — (PNG) [file pcbi.1010764.s025.png]
