# Supplementary material for: Improved visualization of high-dimensional data using the distance-of-distance transformation
Source: PLoS Comput Biol. 2022 Dec 20;18(12):e1010764. doi: 10.1371/journal.pcbi.1010764 (PMC9812310; doi:10.1371/journal.pcbi.1010764)
Supplement: Attachment — Submitted filename: Response to reviewers.pdf [file pcbi.1010764.s027.pdf]

Response to reviewers for: Improved visualization of high-dimensional data using the distance-of-distance transformation

Jinke Liu[1,2] and Martin Vinck[1,2]

[1]Ernst Strüngmann Institute (ESI) for Neuroscience in Cooperation with Max Planck Society, Frankfurt, Germany
[2]Donders Centre for Neuroscience, Department of Neuroinformatics, Radboud University Nijmegen, Nijmegen, Netherlands

July 28, 2022

Contents

1. Review 1 **2**

1.1. MAJOR ISSUES 2

1.2. MINOR ISSUES 12

2. Review 2 **17**

2.1. MAJOR COMMENTS 17

3. Review 3 **20**

3.1. MAJOR COMMENTS 20

3.2. MINOR COMMENTS 23

1. Review 1

Reviewer #1: The paper by Liu & Vinck suggests a method to transform a $N \times N$ pairwise distance matrix such that ”noise” points get smaller distances between each other. As a result, when the transformed distance matrix is used for low-dimensional visualization, such as t-SNE, then all noise points get assembled into one ”island”. This can be convenient as it allows to visually separate noise points. The authors apply their method to spike train recordings and show that it helps distinguish evoked activity and spontaneous activity. I found the method interesting and the experimental demonstrations convincing, and the paper can be a good fit to PLoS Comp Bio. At the same time, I believe major revision is needed to improve the presentation clarity.

We thank the reviewer for these positive comments. Please note that quotations of the manuscript text are in italic, and our responses are in normal font. We note that we have slightly changed the CNN application, by now using images from ImageNet - this did not lead to a change in our conclusion. We have also added an example of using DoD on representations of sketch and natural images (Supplementary Figure S13). We also note that we have removed the UMAP visualization because the manuscript now focuses on t-SNE and explores its parameter settings in more detail.

1.1. MAJOR ISSUES

1. **Intuition paragraph (lines 44-50) is very unclear. Sentence in lines 44-46 – why is that true? Should this be intuitive? It’s written as if this sentence should be self-evident, but I think it isn’t. Line 47: ”to other noise points” – should this be ”to neighbouring noise points”? Is this statement about all noise points or only neighbouring noise points? Line 50: this verbal formulation can be unclear without a formula, I suggest to move the formula and exact definition of your new distance from the Methods here.**

   We thank the reviewer for these comments, which helped us to improve the text. We have now modified our wording in this paragraph to make it more intuitive. Indeed, in line 47, it should be ”to neighbouring noise points” as the reviewer suggested. To illustrate our method more straightforwardly, we moved the formula from the Method section here. We quote here the new intuition paragraph:

   Here, we develop a technique to address the scattering noise problem using a transformation of the distance matrix. We will show that the performance of low-dimensional embedding techniques is often improved by such transformation of the distance matrix. We start from a scenario where there are clusters, but also noise points that are scattering in a high-dimensional space. Consider the distance of a noise point P to its nearest neighbours. In a high dimensional space, we expect another noise point Q to have a similar distance to the nearest neighbours of P as P itself. In other words, even though the distance between two noise points P and Q can be large, their distances to their respective nearest neighbours might in fact be very similar in a high-dimensional space. This is simply due to the fact that scattering points do not have particular clustering patterns, which makes any point almost identical to others in terms of their neighboring distance structure. This observation led us to compute the differences between the distances (i.e. the Distance-of-Distance), such that the scattering nature of noise points can be better captured after the transformation. Specifically, for each pair of data points, we consider the joint set of K neighbours of these two data points, and then compute the Distance-of-Distance w.r.t. this set of neighbour points.

   Mathematically speaking, given a dataset $X \in \mathbb{R}^{N\times D}$ with N samples and D features, the distance matrix is constructed as $\mathcal{D} \in \mathbb{R}^{N\times N}$, where $d_{i,j} = ||X_i - X_j||_1$. For any two data points $X_i, X_j \in \mathbb{R}^D$, we found their sets of K nearest neighbors I and J respectively. Then we manipulated the original distance matrix $\mathcal{D}$ to yield another new distance matrix $\mathcal{F}$. We take the average L1 difference between the two points’ distances to the selected neighborhood as the new distance as following,

   $$f_{i,j} \equiv \frac{1}{2K} \sum_{n\in I\cup J} ||d_{n,i} - d_{n,j}||_1 \quad (1)$$

   Subsequently, the Distance-of-Distance matrix $\mathcal{F}$ is used to substitute the original distance matrix used in the t-SNE algorithm. Applying this transformation, we should be able to obtain a high similarity between noise points, while

retaining the distances from noise points to the clusters, and the distances between the clusters. Moreover, we expect the method to be relatively insensitive to the choice of neighborhood size K. As long as K is kept smaller than the cluster size, the average density of the cluster neighborhood should not change much, therefore, the shrinkage of noise-cluster and noise-noise distances should in general apply (Supplementary Figure S1).

2. **How do the results depend on K? This is never shown. Consider the example in Figure 1 (which is very impressive by the way). What happens if you use K smaller than 10? K larger than 10? K equal to the sample size? This needs to be shown. I am actually unsure whether $K = n$ (where n is sample size) will work or will fail. This is very important, and should be explained in the intuition paragraph (see above).**

We thank the reviewer for this important question. We addressed the issue of neighborhood size in the supporting information as follows:

In the following simulations, we will show that our method is relatively robust to the choice of neighborhood size K as long as K is smaller than the size of the clusters. However, as K approaches sample n, there could be distortions.

We generated 5 clusters following multi-variate Gaussian distributions with different means. The size of each cluster was 20. The data contained another 200 noise points. The feature space had a dimensionality of 50. We found that with K ranging from 5 (smaller than the cluster size) to 20 (equal to the cluster size), the DoD transformation can robustly solve the scattering noise problem (Supplementary Figure S1A). However, in situations where there are more clusters, with K further approaching the total number of points n, the DoD transformation introduced distortions to the clusters and failed to put scattering noise points together (Supplementary Figure S1B).

To explain this, consider two points from two distinct clusters. As K becomes very large, the sets of neighbours of these two points will start including more distant noise points. Because of this, the Distance-of-Distance between these cluster points will strongly shrink. Consequently, the advantage of the method is lost, because the shrinkage of the cluster and the noise points becomes comparable. Thus, it is generally desirable that K is not too large (ideally around or smaller than the cluster size).

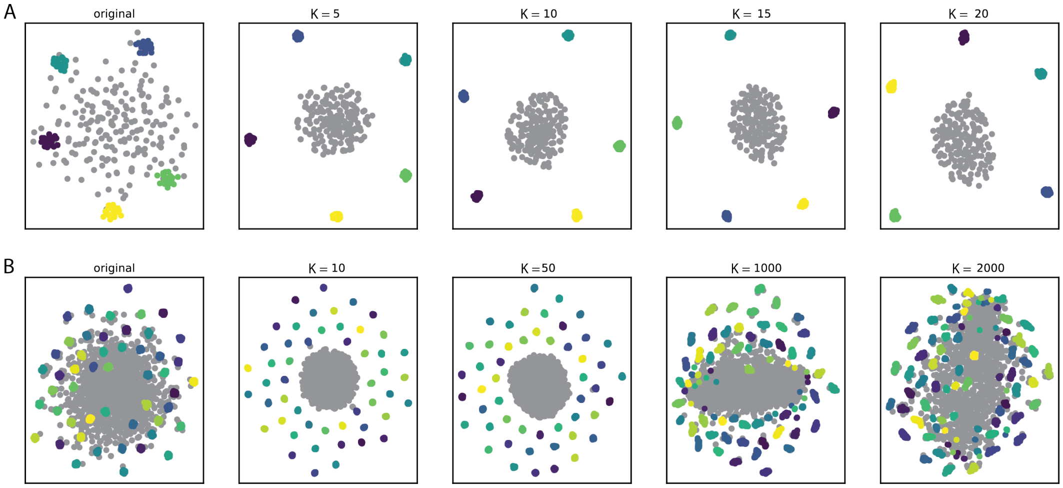

Figure S1: Effect of neighborhood size K. A. 5 clusters, each with 20 points; 200 scattering noise points; dimensionality of 50; Original embedding (left) and DoD transformation with a neighborhood size ranging from 5 to 20. **B.** 50 clusters, each with 20 points; 1000 scattering noise points; dimensionality of 50; Original embedding (left) and DoD transformation with a neighborhood size of 10, 50, 1000 and 2000.

We also added an explanation to the intuition paragraph accordingly:

Moreover, we expect the method to be relatively insensitive to the choice of neighborhood size K. As long as K is kept

smaller than the cluster size, the average density of the cluster neighborhood should not change much, therefore, the shrinkage of noise-cluster and noise-noise distances should in general apply (Supplementary Figure S1).

3. **Are there any downsides of using the transformation? This is never discussed, but should be. In particular, what happens with the noise-free dataset if it is transformed? The authors suggest that nothing much would change, but they need to show direct evidence/quantification. What if the noise-free dataset has some continuous structures? What if one of the clusters has low density (but is still well isolated from the other clusters)? Can you take some real-world noise-free data and show t-SNE embeddings before/after the transformation?**

   We have performed several additional analyses to address this point. We find that in general, with a reasonable neighborhood size, the DoD transformation method does not introduce distortions to the embeddings in the absence of noise points. These results are shown in the supporting material:

   Next, we quantify the downsides of applying DoD transformation in the absence of scattering noise points in the dataset. Firstly, we applied the DoD transformation to a simulated noise-free dataset and examined whether the relative positions across the cluster centers were maintained. We found that the separation of clusters is maintained for a small K. For K larger than the cluster size, though we observed some distortions in the shape of clusters, the separation between clusters was largely preserved, as it is indicated by the high correlation coefficient between dissimilarity matrices calculated from the low-dimensional embeddings (Supplementary Figure S6). Similarly, we generated a noise free data set where one of the clusters had a lower density (Supplementary Figure S7). We found that the separation of clusters was improved for both small and large K. However, the exact location of the clusters show variability, which is due to t-SNE itself based on the random seed used for initialisation [1].

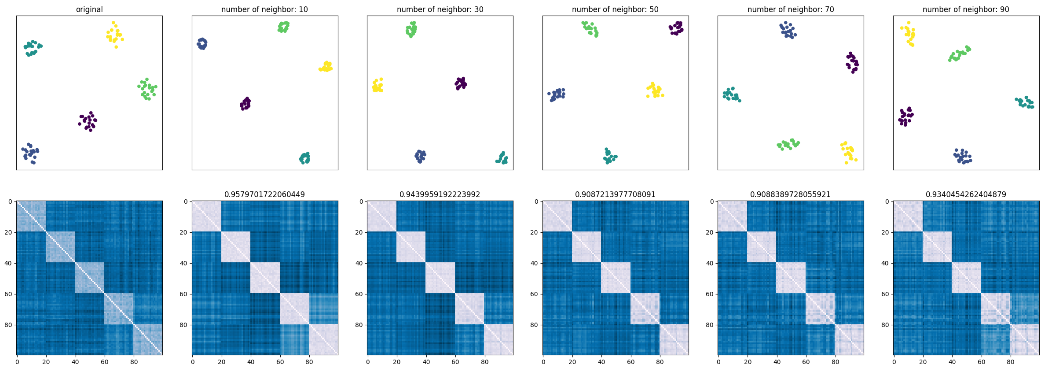

Figure S6: DoD transformation does not distort the clustering. 5 clusters, each with 20 points; dimensionality of 50; DoD transformation with neighborhood size ranging from 5 to 30.

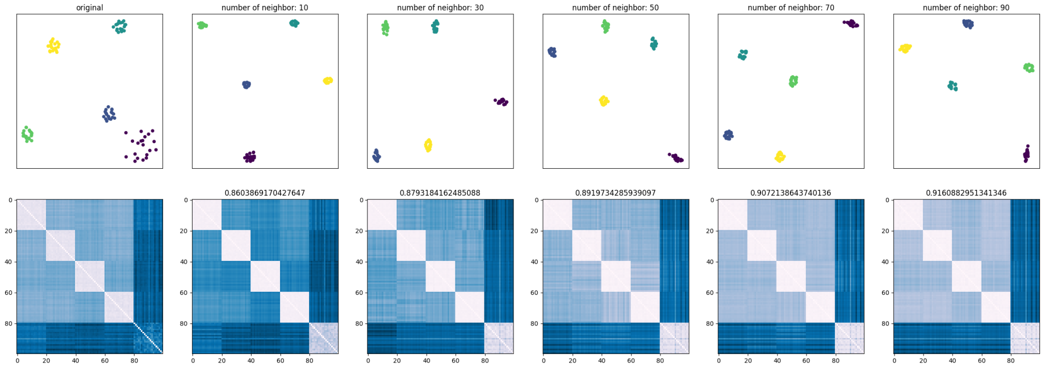

Figure S7: DoD does not distort the clustering with one class of low density. 5 clusters, each with 20 points; dimensionality of 50; DoD transformation with neighborhood size ranging from 5 to 30. All clusters were generated from multivariate Gaussian distribution and one of them is with a larger standard deviation (0.5) than the rest (0.2).

To investigate whether the DoD transformation may distort the continuous structure in data in the absence of noise, we analyzed a calcium imaging data set from mouse V1 [2]. Mice were shown gratings with a total of 32 different stimulus directions. By visualizing the neural activities in a 2D embedding, it can be observed that the trials are organized in a continuous ring structure. This is expected as stimulus direction is a circular variable. After applying DoD transformation on the distance matrix, we can see that the ring structure was largely maintained (Supplementary Figure S10). The distance matrices had a similar structure and were highly correlated ($\rho = 0.875$). In addition, we trained a KNN classifier on both the original neural representations and on the 2D embeddings. We found that after DoD transformation, the cross-validated performance decreased only from 94.3% to 91.8% on high-dimensional neural representations, and it decreased only from 88.7% to 87.4% on the low-dimensional embeddings. This suggests that DoD transformation introduces very limited distortions and preserve the geometry in the original manifold, even for a continuous structure.

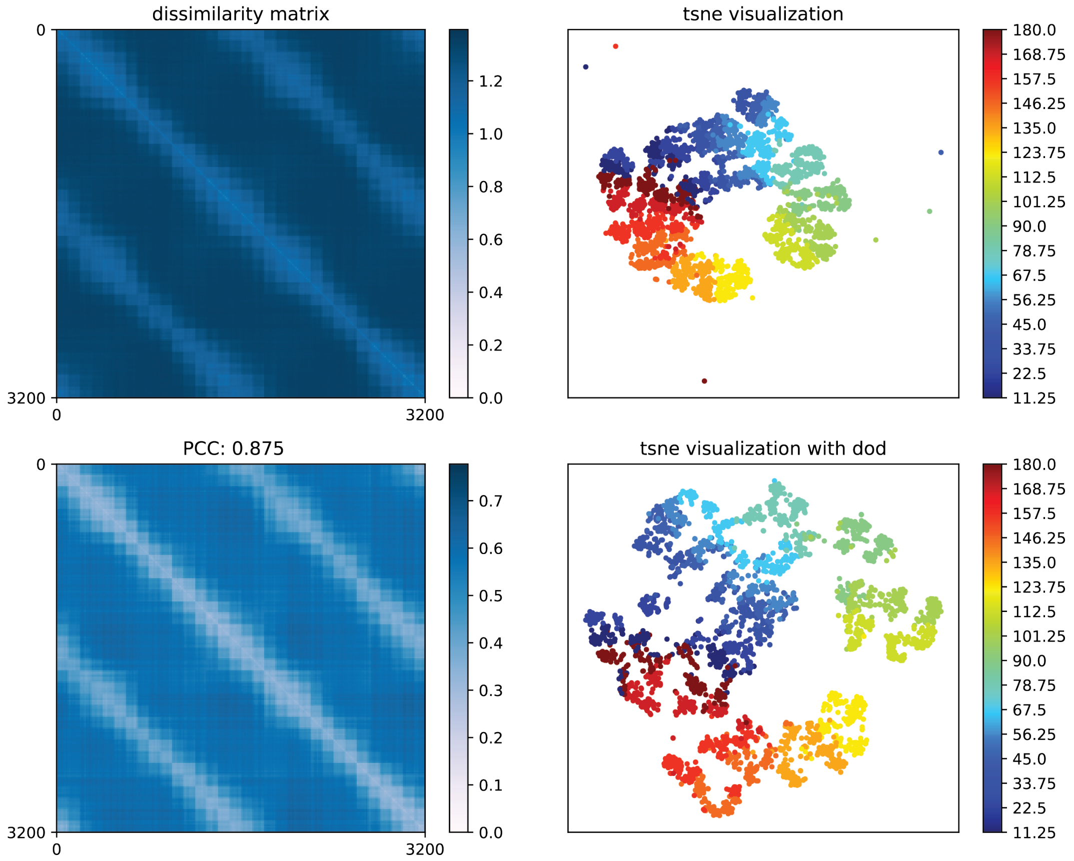

Figure S10: DoD transformation keeps the ring structure of neural representations of grating stimulus. Each data point represents the populational firing pattern in a given trial ($n = 3200$). Trials with different stimulus orientations are labelled in different colors.

4. **Continuing previous point: this can be done in Figure 6. If you apply your transformation to the data in panel A, do t-SNE/UMAP become worse or not? You could show it. You could also quantify it by computing kNN classification accuracy, not only in the t-SNE/UMAP embedding, but also directly based on distances (before t-SNE).**

   As it is shown in the previous point, with a modest size of neighborhood, the geometry of noise-free data in both neural spiking data example (Supplementary Figure S11) and in convolutional neural network example (Supplementary Figure S12) was largely maintained after applying the DoD transformation. These results are shown in the supporting material:

   Next, we applied the transformation to neural spiking data as in Figure 5 but without spontaneous activities. We found that the separation between clusters corresponding to different stimulus orientations increased with the DoD transformation (Supplementary Figure S11). This result suggests that DoD transformation can improve the clustering even for a noise-free dataset.

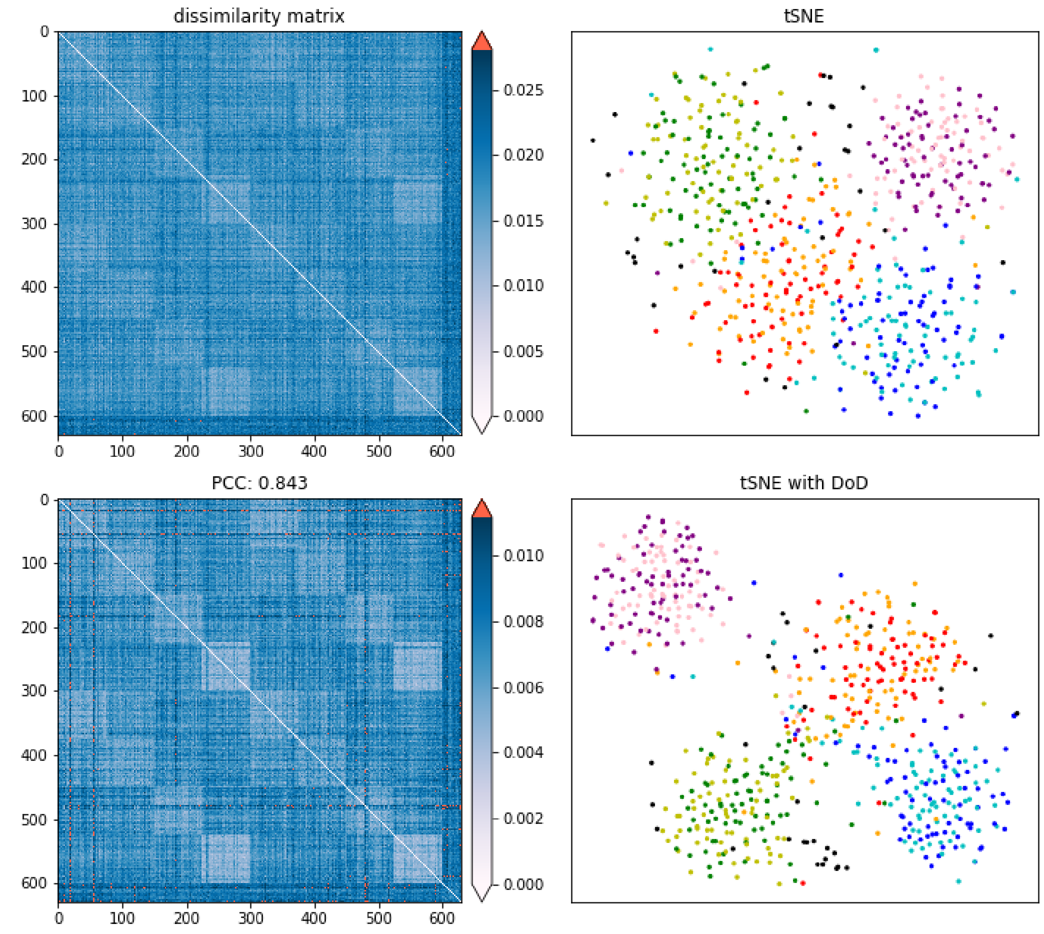

Figure S11: Neural manifolds were better separated by DoD transformation. Each data point represents the populational spiking pattern in a given trial ($n = 600$). Trials with different stimulus orientations are labelled in different colors.

To further test whether DoD transformation can alter the geometry of noise-free real data, we applied the transformation to convolutional neural network representations of image patches. We found that with a modest neighborhood size ($K = 5$), the relationships between clusters of different objects was largely maintained. We found that after DoD transformation, KNN classification performance increased slightly from 98.8% to 98.9% on high-dimensional neural representations, and it decreased slightly from 98.7% to 98.5% on the low-dimensional embeddings (Supplementary Figure S12).

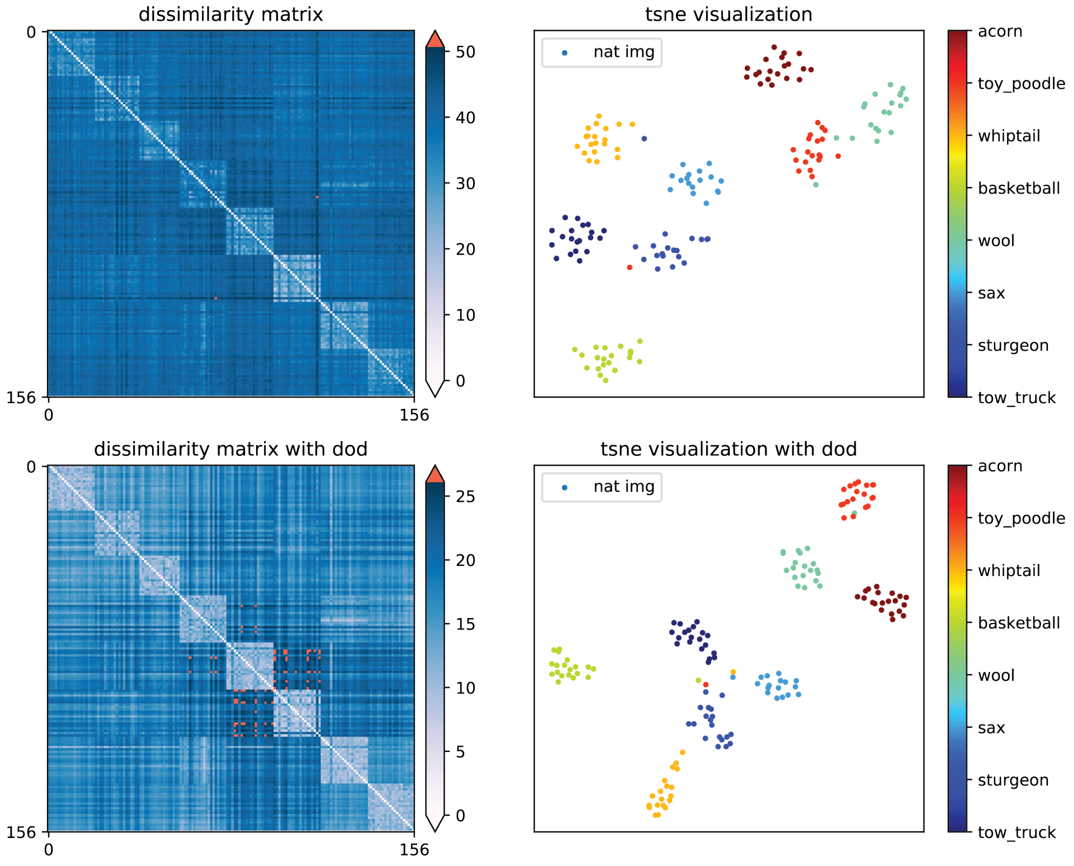

Figure S12: Object manifolds were not distorted by the DoD transformation. 2D t-SNE embeddings for 8 random ImageNet classes maintain clustering patterns after the DoD transformation.

5. **Figure 1 and subsequent simulation Figures (e.g. Figure 2) always have many more noise points than non-noise points. Will the algorithm work as well if noise points are only a small fraction of the total sample size? Please show that.**

   In situations where there are few scattering noise points, DoD transformation still managed to work. We included the discussion in the supplementary information:

   In situations where there are less noise than the number of clusters, DoD transformation still succeeded in putting all noise points as a separate cluster (Supplementary Figure S4).

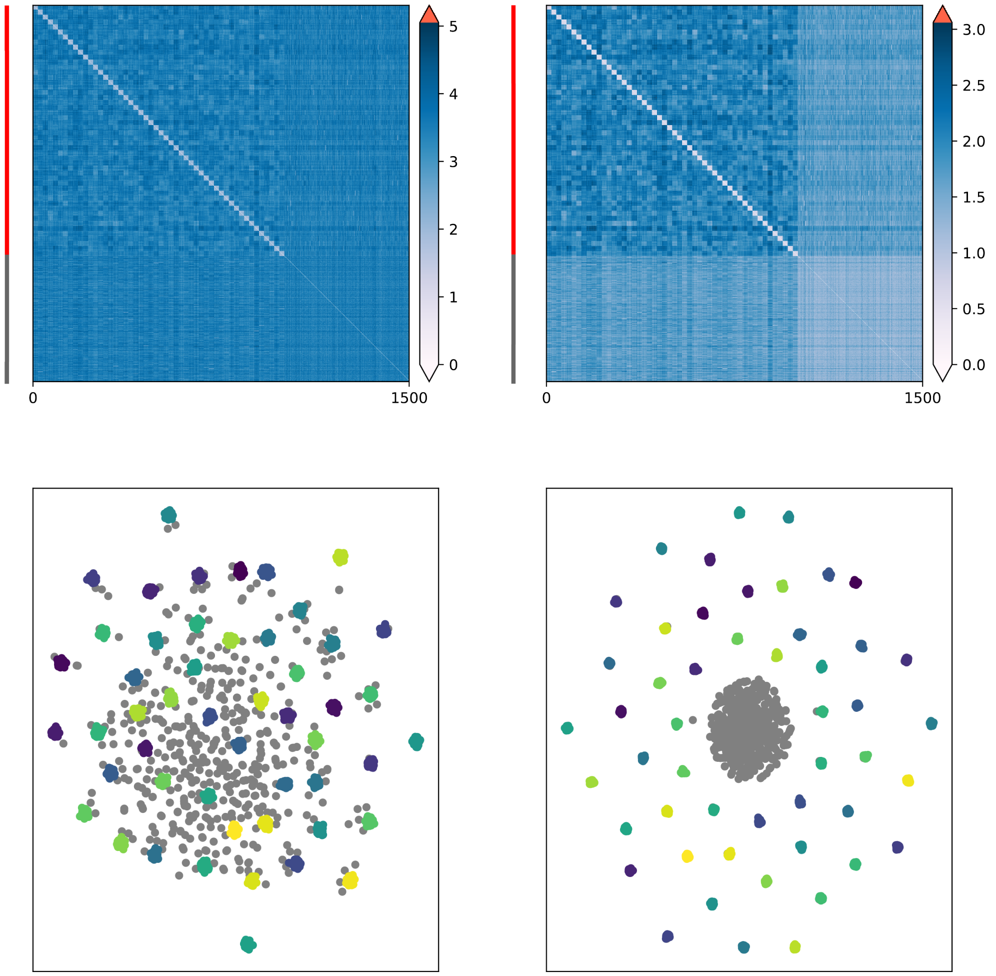

Figure S4: DoD transformation keeps its performance when there are less noise points. 50 clusters, each with 20 points; 500 noise points; dimensionality of 50; DoD transformation with a neighborhood size of 5. *Left*, original t-SNE visualization. *Right*, t-SNE visualization with DoD transformation.

6. **line 92: can variance be approximated as well? Using the variance could strengthen the analysis.**

   This is an interesting question, however we did not directly see the relevance of estimating the variance or the way it depends on the DoD transformation.

7. **line 188: "clearer separation" – is this some t-SNE effect or a real effect in the distances matrix? To answer that, you could use kNN classification accuracy, or do before/after t-SNE embeddings of only evoked response points.**

   We trained KNN classifier on both the high-dimensional distance matrix and low-dimensional embeddings. The cross-validated classification accuracy indicates that with DoD transformation, the data points were more separated and

clustered. We included this discussion in the supporting information:

We show that KNN classification accuracy on the stimulus-evoked responses was higher for Distance-of-Distance matrices even without t-SNE (Supplementary Figure S9). Here, we trained the KNN on both original high-dimensional data and t-SNE embeddings with the presence of noise. In both cases, the average cross-validated accuracy on the test dataset was higher.

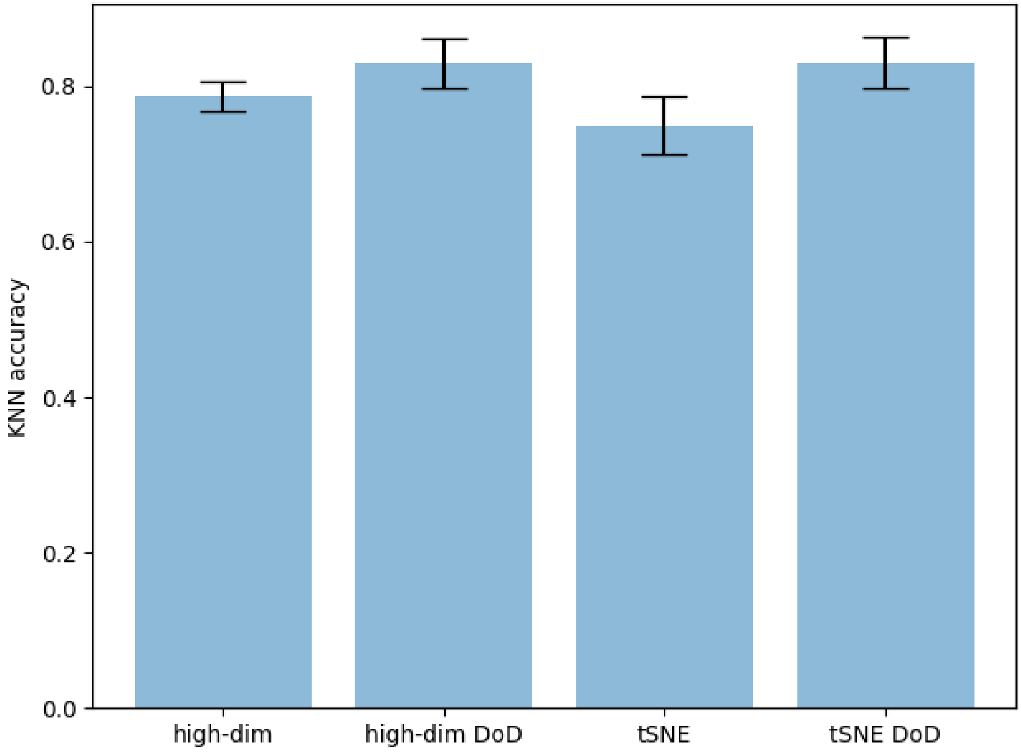

Figure S9: DoD transformation improves KNN classification of neural spiking sequences. For both the distances in the high dimensional space and the distances in the low dimensional embedding, the cross-validated KNN classification scores are higher after DoD transformation.

8. **Most of the Methods (sections 4.1-4.2) is textbook description of t-SNE. Frankly, it can be removed, or condensed to a small paragraph.**

   We thank the reviewer for the suggestion. The textbook description of t-SNE algorithm is now removed.

9. **Section 4.3 should be moved to Results, it basically consists of 1 formula. The notation is very confusing! D is used for dimensionality and also to denote distance matrix. k was called K before. Why two notations for the set of nearest neighbors? You don't need to define nearest neighbors, so formulas 18 are unnecessary. $\mathcal{N}$ is never defined. In equation 19, do you divide by $2k$ or by the size of the $\mathcal{I} \cup \mathcal{J}$? The sum should be over union of $\mathcal{I}$ and $\mathcal{J}$ which is not denoted by $\{\mathcal{I}, \mathcal{J}\}$.**

   We fixed the mathematical notation in Equation 2 to make it less confusing. In DoD transformation, we have the same neighborhood size for each point in a given pair, therefore the size of the joint set should be $2K$, i.e. $2K = |I \cup J|$.

Mathematically speaking, given a dataset $X \in \mathbb{R}^{N \times D}$ with N samples and D features, the distance matrix is constructed as $\mathcal{D} \in \mathbb{R}^{N \times N}$, where $d_{i,j} = ||X_i - X_j||_1$. For any two data points $X_i, X_j \in \mathbb{R}^D$, we found their sets of K nearest neighbors I and J respectively. Then we manipulated the original distance matrix $\mathcal{D}$ to yield another new distance matrix $\mathcal{F}$. We take the average L1 difference between the two points' distances to the selected neighborhood as the new distance as following,

$$f_{i,j} \equiv \frac{1}{2K} \sum_{n \in I \cup J} ||d_{n,i} - d_{n,j}||_1 \tag{2}$$

Subsequently, the Distance-of-Distance matrix $\mathcal{F}$ is used to substitute the original distance matrix used in the t-SNE algorithm.

10. **Can the distance matrix after transformation be used for clustering?**

    Yes. In Figure 4 we showed that using the distance matrix after DoD transformation, we can achieve better K-means clustering performance.

11. **Can your method somehow infer which of the points actually corresponded to noise? Take Figure 1: imagine I don't know that grey points are noise. Can the algorithm identify that?**

    Yes, with a simple computation, DoD transformation can be used to infer the identity of the unlabelled points, thus, it can serve as a denoising method. We added the discussion to the supplementary information:

    DoD transformation can be used as a noise detection method. This is based on the fact that after DoD transformation, the noise point cloud no longer scatters and its density increases. Therefore, we can use K-means or hierarchical clustering on the Distance-of-Distance matrix to identify the clusters. Then, we compute how much the within-cluster distances change after DoD transformation. Because the density changes more for noise clusters than data clusters, the noise cluster can be identified as the group of points that shows more within-cluster distance change.

    Moreover, even without clustering, we can find scattering noise points by directly investigating how the set of neighbors changes for each point after the transformation. The neighborhood of a noise point changes more compared to a non-noise point. Thus, the identity of neighbours changes more for noise points than for cluster points after the DoD transformation. In our simulation, we measure the changes of neighbor identities as the overlap rate of neighborhood before and after the DoD transformation. A larger overlap rate indicates a smaller change. We find that the distribution of neighborhood overlap rate exhibit bi-modality. By using a mid point method, we could calculate a threshold to bipartite the distribution. We find that the population with smaller neighborhood overlap rate can be identified as noise points (Supplementary Figure S2).

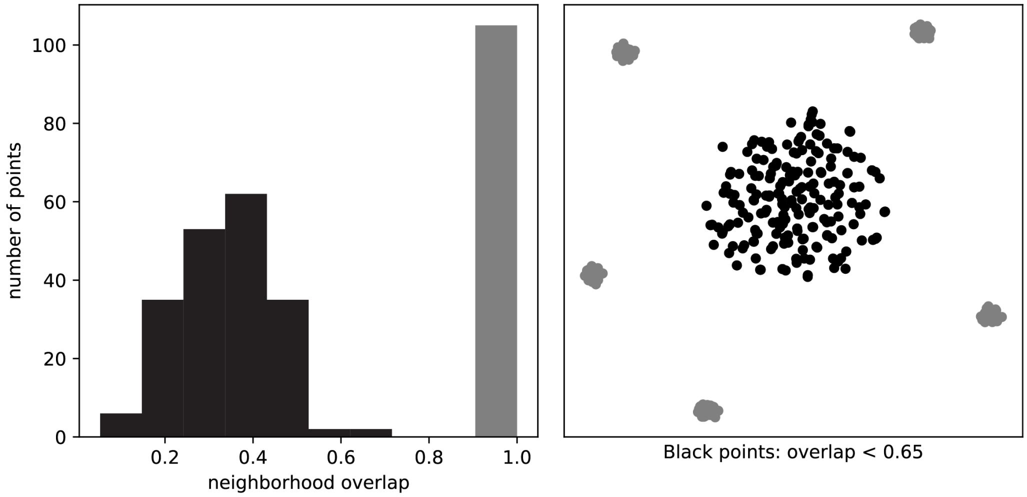

Figure S2: Inference of noise points based on neighborhood overlap. A. Distribution of overlap rate of neighborhood identity before and after the DoD transformation. **B.** Points with smaller overlap rate ($< 65\%$) were identified as scattering noise points (black).

1.2. MINOR ISSUES

1. **Ref [4] is a strange choice in line 8. This paper does use t-SNE but it's a very minor application there, while scRNA-seq literature has a lot of papers that use t-SNE/UMAP more prominently, to visualize much more complex datasets.**

   We thank the reviewer for the suggestion. We include more relevant literature that applies t-SNE in scRNA-seq field such as [1, 3–5].

2. **Introduction is too short, sloppy in places, and does not give literature overview:**

   (a) **What is "density-based" in line 20? How is t-SNE density-based?**

   We agree with this point and have removed this wording.

   (b) **line 22: "maximally preserving pairwise distances" – sloppy. t-SNE does not aim to preserve distances at all.**

   We agreed that t-SNE does not try to preserve the distances but only attempts to preserve the neighboring relationships. This sentence has now been removed.

   (c) **"crowding problem" – unfortunate terminology, as the original 2008 t-SNE paper uses the same term "crowding problem" to refer to something else!**

   We thank the reviewer for pointing this out. In order to prevent further confusion, we refer to it as the "scattering noise problem".

   (d) **there are too few references. Are there any related papers at all? That deal with noise in the data? If not in the context of dimensionality reduction, then maybe clustering? If you cannot find any relevant research whatsoever, at least say so.**

The problem of noise is known in clustering, e.g. HDBSCAN assigns points as noise that are not assigned to any cluster, based on the output of the minimum spanning tree [6]. However at the level of low-dimensional visualization, this would not resolve the scattering noise problem. This has now been mentioned in the introduction section.

We have updated the Introduction of the manuscript:

A major goal of data science is to extract patterns from high-dimensional data containing multiple features. It is typically required to construct a low-dimensional representation of high-dimensional data for the purpose of visualization, noise reduction, or feature extraction. In fields such as biology, where high-dimensional data sets are common, dimensionality reduction approaches are widely adopted. For instance, in neuroscience, dimensionality reduction techniques have been used to study the way in which neuronal populations represent motor and visual information [2, 7, 8]. It is also a standard approach to study the the genetic profiles of different cell types [1, 3–5]. Dimensionality reduction techniques based on embeddings including t-SNE [9, 10] and UMAP [11] have been developed to represent high-dimensional data with only two or three components. The principle underlying these techniques is to treat data points as particles that are attracted to their neighbours and repelled by distant data points. Despite their usefulness, it is known that algorithms like t-SNE have inherent limitations, such as: sensitivity to hyper-parameters like perplexity; difficulty to capture global structure in the data especially when there are many clusters [12]. Therefore, it is important to optimize the pre-processing of the data and application of low-dimensional embedding techniques [1].

Here we show another problem with methods like t-SNE, namely that their performance strongly deteriorates when the data set contains many noise points. We show that the low-dimensional embedding space can become crowded due to the presence of noise points (i.e. points falling outside clusters). The basic mechanism is that noise points repel each other and therefore start overlapping with clusters, even though the noise points have large distances to the clusters. As a result, meaningful patterns in the data can be masked. To our knowledge, there exists no simple solution to this "scattering noise problem". Although clustering techniques like HDBSCAN can be used to identify noise points [6], these techniques do not solve the scattering noise problem in terms of low-dimensional visualization. Furthermore, although in some situations PCA may aid to denoise the data, PCA can also remove information from high dimensional data sets. As we will show, PCA does not in general effectively solve the scattering noise problem.

Here, we present a simple technique to solve the scattering noise problem. We show that scattering noise problem for high-dimensional datasets can be effectively alleviated with a transformation of the distance matrix. We call this the Distance-of-Distance (DoD) transformation, because it considers the differences between distances in a certain neighborhood of the data points. We apply the Distance-of-Distance transformation to both electrophysiological recordings of neurons in the mouse visual cortex during the presentation of drifting grating stimuli, as well as representations of natural image patches by convolutional neural network units. We demonstrate that in both cases, the Distance-of-Distance transformation facilitates the separation between noise points and cluster points in the low-dimensional embedding space.

3. **line 58: "data poits" → "noise data points"?**

   We thank the reviewer for pointing this out. This has been fixed now.

4. **Figure 2: add column titles indicating dimensionality.**

   We thank the reviewer for the suggestion, more illustrations were added on the top of Figure 2 to indicate the dimensionality.

5. **line 82: K was used for number of neighbors, now it's number of clusters – confusing**

   We thank the reviewer for pointing out this mistake. We changed the notation for number of clusters into M. Accordingly, the set of m-th cluster points is denoted as $\mathbb{C}_m$.

6. **line 111: "while preserving distance between the clusters and the noise points" – that's sloppy formulation, these distances are not exactly preserved.**

We agree with the reviewer that these distances are not exactly preserved. As it is illustrated in Figure 3, distance between cluster points and noise points are less affected and relatively more preserved. The wording of this sentence has now been changed.

7. **Figure 3: what are error bars?**

   Error bars indicate the standard deviation across simulations with different initialization conditions. This piece of information was now added to the caption.

8. **Figure 3: unclear what is the cluster variance used for GMM simulation – it should be some realistic data, not infinitely dense.**

   The cluster variance used for GMM simulation is 0.1, which is small but not 0 as infinitely dense. More details of the simulation can be found in the method section.

9. **Figure 5, panel B: y-axis ticks are cropped**

   This figure has now been fixed.

10. **Figure 5, panel C: could you label "signal" and "noise" parts of the distance matrix?**

    We added more illustrative labeling of "signal" and "noise" parts of the distance matrix for all figures. Red bars on the left indicate "signal" part while black bars indicate "noise" part.

11. **Figure 5, panel D: what are the black points?**

    These points are stimulus epochs whose stimulus information is missing due to poor labelling. In these epochs, no drifting grating stimuli were shown, so they are essentially spontaneous activities. This illustration was added to the caption now.

12. **line 204: please mention some memory and runtime requirements. How long does it take for what sample size?**

    We included the runtime discussion in the supplementary information:

    We can see in Supplementary Figure S14 that the run time of DoD transformation increases exponentially with the number of data points N. A larger neighborhood size could also increase the run time a bit, especially when the distance matrix is large. And as it is shown in Figure 3C/D as the number of data points N increases, the effectiveness of DoD transformation decreases. With a fixed dimensionality D, larger the dataset size, less effective the transformation and larger the time cost. These disadvantages can only be compensated if the data points are living in a higher dimension.

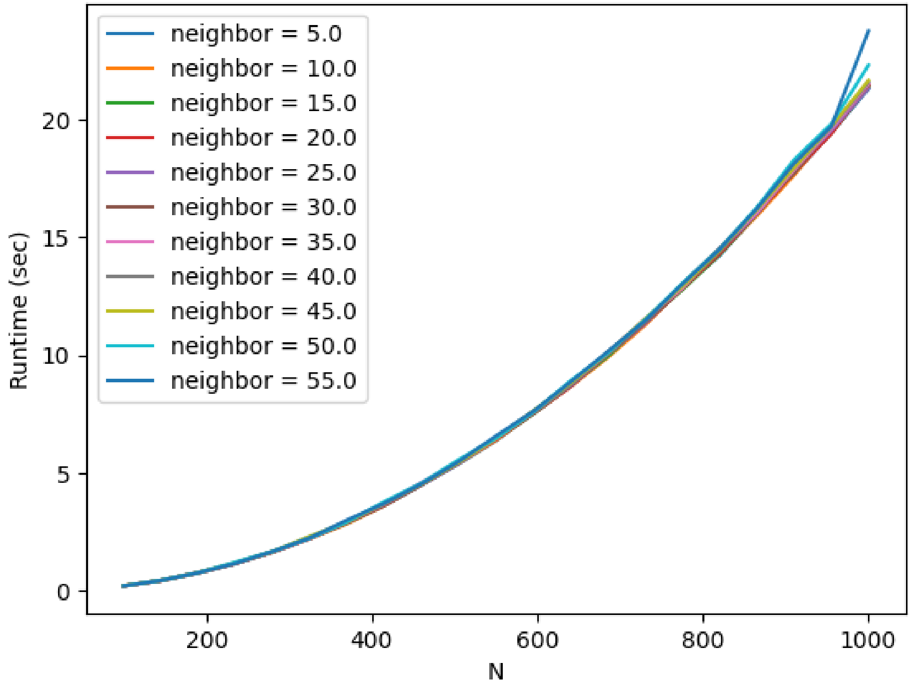

Figure S14: Runtime of the DoD transformation

13. **line 206: "further work is needed..." – is this at all feasible? Sounds rather unfeasible to me.**

    We agree that this is a bit speculative and have removed this part.

14. **line 210: "distortions" – this needs to be shown, see my comment above about choice of K**

    With DoD transformation, distortion happens with a large neighborhood size. We addressed it in the supplementary materials as mentioned previously (Supplementary Figure S1).

15. **Section 4.4: what t-SNE implementation was used? Give version. And UMAP?**

    We included more details in the method section:

    We generated high-dimensional cluster points and noise points by randomly sampling from the multivariate Gaussian distributions with a standard deviation of 0.1. We drew points within one cluster from the same Gaussian distribution, while noise points were independently distributed. Then, we calculated the distance matrix between all pairs of data points by using either euclidean or city-block metric. To find the low-dimensional embeddings, we applied t-SNE algorithm with perplexity value ranging from 5 to 50 and initialization with different random seed. We used an open implementation of t-SNE algorithm from sklearn (version 0.23.2). In some settings, we adopted PCA initialization combined with PCA preprocessing (Supplementary Figure S3).

    To analyze how dimensionality and number of points influenced the performance of Distance-of-Distance transformation, we simulated cluster points from Gaussian distributions with small variance and noise points randomly distributed in a hyper-cube. Next, to compute the cluster-to-cluster distance, we considered all pairs of points, each from a different cluster, then we calculated the average distance of all such pairs. To compute the cluster-to-noise distance, we considered all pairs of points, such that one is a noise point and the other from a cluster, and then calculated the average distance between all such pairs. To compute the noise-to-noise distance, we considered all pairs of noise

points, then we calculated the average distance of all such pairs. Next, we used Distance-of-Distance transformation to manipulate the original pairwise distance matrix $\mathcal{D}$. After the manipulation, we obtained the distance of distance matrix $\mathcal{F}$. We measured the distance shrinkage of all three types of distances (cluster-to-cluster, cluster-to-noise, and noise-to-noise) in two ways: Either the absolute shrinkage was calculated as $\Delta = \bar{d} - \bar{f}$, or the fraction was calculated as $\bar{f}/\bar{d}$. Therefore, a larger delta distance or a smaller fraction indicates a larger shrinkage. In order to measure the clustering performance, we adopted the commonly used metric Adjusted Rand Index (ARI). Corresponding code is available at Github.

16. **line 271: "different initialization" – what initialization?**

    We now included more sufficient details about initialization in the method section. Please see previous point.

17. **line 273: "small variance" – this is insufficient level of detail. Please describe all your experiments EXACTLY. Make it clear what refers to what experiment (which figure) exactly.**

    We now included the exact experimental settings needed to replicate the simulation. We quote from the updated method section. Please see previous point.

2. Review 2

Reviewer #2: This is a manuscript on improving dimensionality reduction through an intermediate transformation of data that filters noise by essentially condensing noise points so that they do not clutter data that represent signals. It is an interesting and relatively straightforward improvement that will be applicable to data that exist in high dimensions with a moderate number of points (the authors state that its improvements slow down with larger N). The authors do a good job of constructing the idea from first principles, creating an appropriate simulation, and finally applying the method to real data. The authors have not provided a link to the code and data repositories, though I assume that they are awaiting review before making their code public.

We thank the reviewer for these positive comments. Please note that quotations of the manuscript text are in italic, and our responses are in normal font. We note that we have slightly changed the CNN application, by now using images from ImageNet - this did not lead to a change in our conclusion. We have also added an example of using DoD transformation on representations of sketch and natural images (Supplementary Figure S13). We also note that we have removed the UMAP visualization because the manuscript now focuses on t-SNE and explores its parameter settings in more detail.

2.1. MAJOR COMMENTS

1. **Please provide a link to code/data repositories**

   We provided the Github link as follows in the method section Github.

2. **The UMAP minimum_distance parameter (particularly low minimum distance) and the t-SNE parameter perplexity can address the tightness of clusters in low dimensions. This can help separate clusters. It would be worth it to run the data from Figure 4A again with a higher perplexity as a sanity check (I suspect your transformation will still be better)**

   We have removed the discussion of UMAP to focus on t-SNE improvement. Perplexity controls how much focus t-SNE puts on the global structure. Though perplexity influences the geometry of the low-dimensional embedding, the ability of DoD transformation to separate scattering noise was unaffected. We illustrated this point in the supplementary section:

   Perplexity is an important tunable parameter for the t-SNE algorithm. In the original t-SNE paper, the author suggested that the choice of perplexity value within a reasonable range between 5 and 50 did not seem to influence the experiment results [9]. However, in practice, it has been suggested to use 1% of the total sample size of the dataset [1]. Different choices of perplexity change the behavior of t-SNE [12]. To test whether the scattering noise problem can be solved by t-SNE algorithm itself, we carefully tuned the perplexity value based on the number of points in each cluster and the number of clusters. However, we did not observe the alleviation of the "scattering noise problem". With a higher perplexity value, some local geometrical structures may emerge in the Gaussian clusters as they became more ellipsoidal and also in the random noise cloud. However, we observed that there existed no perplexity value that could make noise points attracted to each other, and the scattering noise problem remained (Supplementary Figure S5).

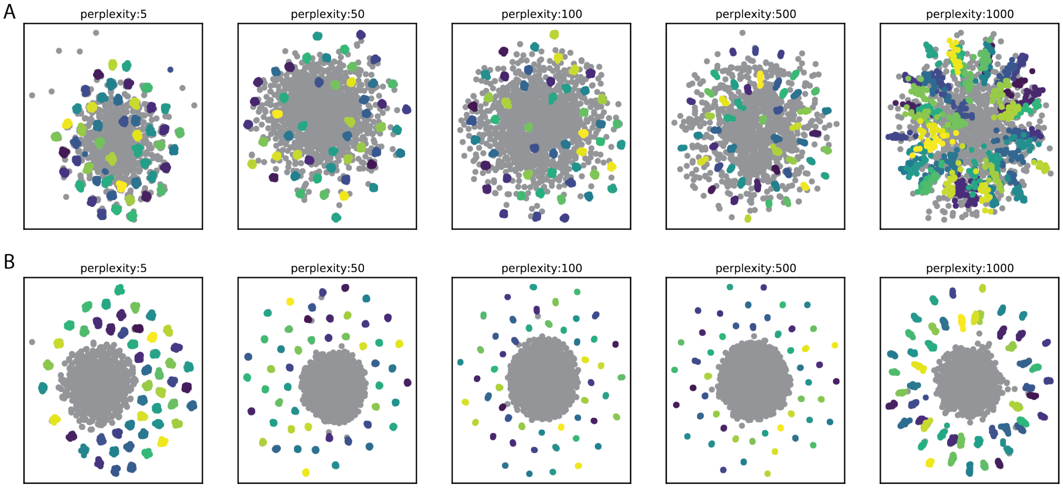

Figure S5: Larger perplexity of t-SNE algorithm does not solve scattering noise problem. 50 clusters, each with 20 points; 1000 noise points; dimensionality of 50; DoD transformation with a neighborhood size of 5; perplexity values are 5, 50, 100, 500, 1000 from left to right. **A.** t-SNE on original distance matrix. **B.** t-SNE on distance matrix after DoD transformation.

3. **"Drifting grating" is not defined – for those unfamiliar with neuronal stimulus research a short description (e.g. "moving bars") would be helpful.**

   We included more description of the visual stimulus in the method section:

   The drifting grating visual stimulus consists of a full-field sinusoidal grating that moves in a direction perpendicular to the orientation of the grating. The spatial-temporal frequency of the drifting grating stimulus is not considered in our study. In the public dataset provided by Allen Institute, the drifting grating stimulus moves in 8 different directions. They were shown to the animal in a random order (Supplementary Figure S8A).

4. **The labels for scale bars on most heat maps are confusing because of spacing between the plots and scales. Figure 4C is clear, however, and is a good template for the other figures.**

   We thank the reviewer for the suggestion. Now the colormaps for all the distance matrices were replaced to show clearer patterns.

5. **For Figure 5, the spontaneous activities in gray are exemplified by the raster plot in 5B, correct?**

   Yes, raster plot in Figure 5B is an example of spontaneous activities during the inter-trial interval. And in Figure 5D, all the spontaneous activities are denoted as noise points in grey.

6. **In your discussion you mention that the algorithm is computationally intensive since it calculates an NxN distance matrix. Providing runtimes for some of the transformations that you carried out would be very useful.**

   We include the discussion on runtime as a supplementary figure.

   We can see in Supplementary Figure S14 that the run time of DoD transformation increases exponentially with the number of data points N . A larger neighborhood size could also increase the run time a bit, especially when the distance matrix is large. And as it is shown in Figure 3C/D as the number of data points N increases, the effectiveness of DoD transformation decreases. With a fixed dimensionality D, larger the dataset size, less effective the transformation and larger the time cost. These disadvantages can only be compensated if the data points are living in a higher dimension.

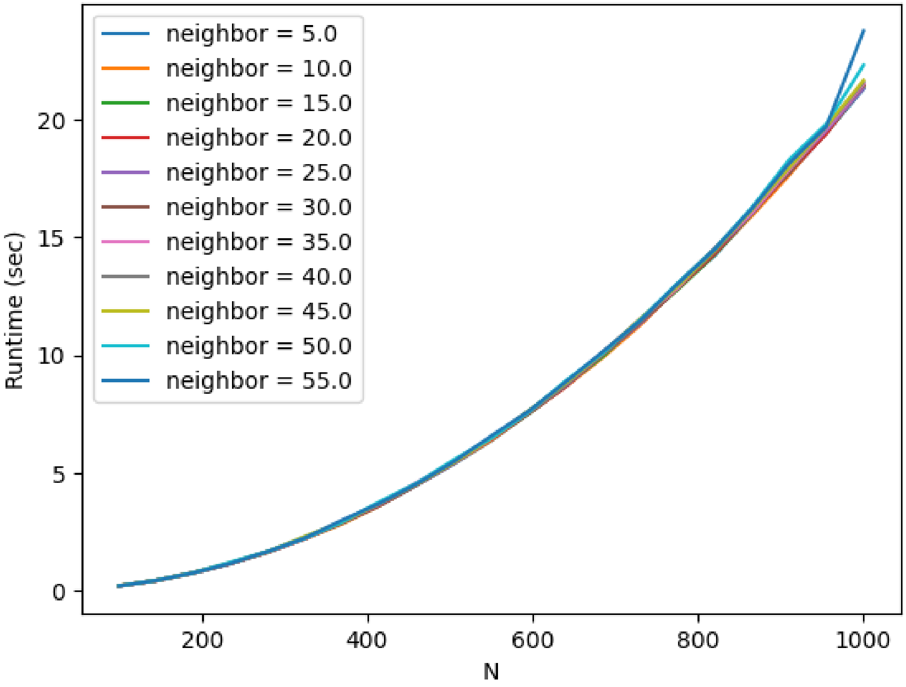

Figure S14: Runtime of the DoD transformation

7. **Is there a point at which you would consider this transformation's tradeoffs to be too onerous? I'm referring specifically to Figure 3C/D, where large N reduces the effectiveness of this transformation, as well as your comments on the computational intensity of transformation of an NxN matrix in D dimensions.**

   Like the reviewer suggested, as the number of data points N increases, the effectiveness of DoD transformation drops and the runtime cost increases. Therefore, it is important to keep in mind that such transformation is most effective when applied to a high-dimensional data set (see previous point).

3. Review 3

Reviewer #3: In this paper, Liu and Vinck describe an interesting strategy for obtaining more informative 2D embeddings of high-dimensional data. The strategy is simple: convert the distance matrix typically used by embedding methods into a Distance of Distances matrix. This appears to be very useful on two real-world datasets, and the simulated data also drives the point home. I have two major concerns for this manuscript.

We thank the reviewer for these positive comments. Please note that quotations of the manuscript text are in italic, and our responses are in normal font. We note that we have slightly changed the CNN application, by now using images from ImageNet - this did not lead to a change in our conclusion. We have also added an example of using DoD transformation on representations of sketch and natural images (Supplementary Figure S13). We also note that we have removed the UMAP visualization because the manuscript now focuses on t-SNE and explores its parameter settings in more detail.

3.1. MAJOR COMMENTS

1. **There is an automatic rescaling of distances in t-SNE/UMAP based on the number of neighbors each point has, via the perplexity parameter. This should take care of the relative density problem that this paper deals with. I would like to make sure that this automatic rescaling was enabled, and that the authors always determined a separate σ_j for each point. However, the code was not shared, so I cannot check this directly. Furthermore, there are many Euclidian distances presented to us throughout the manuscript, which makes me think the distances were not re-estimated. Furthermore, the theoretical analyses indeed do not consider the rescaling of distances implicit in t-SNE/UMAP.**

   If σ_i was indeed estimated directly for each point as it should be, then I would like to know why the authors think that was not enough. If it was estimated, then I think a lot of the visualizations should change to show the transformed similarity matrices instead of the Euclidian distances, since the transformed similarity matrices are the actual data that t-SNE uses. The theoretical analysis itself probably has to be redone to introduce the σ_j's in section 2.1. Confusingly, sigma here takes a different meaning, so that variable name has to change too.

   I am not sure how you are running t-SNE, but if you're passing in a similarity matrix directly to an open implementation of t-SNE, then you would have needed to do the point-by-point rescaling by yourself. There is way too little information in the methods about what you did, so please add all those details there as well. There are some other important parameters in t-SNE and UMAP, you need to specify those in your Methods section.

   We used an open implementation of t-SNE algorithm from sklearn. With the "precomputed" option, we can pass in any precomputed dissimilarity matrix and the point-by-point rescaling was carried out by the algorithm to estimate the probability of neighborhood (the method is called _joint_probabilities in sklearn implementation). More specifically, we calculated the Euclidean distance matrix D between any two d-dimensional data points $D_{ij} = ||X_i - X_j||_2, X_i, X_j \in \mathbb{R}^d$, and passed it to the t-SNE algorithm implemented by sklearn. And the conditional probability of observing i given a Gaussian distribution centered on j is calculated as,

   $$P_{i|j} = \frac{\exp\left(-\frac{D_{ij}^2}{2\sigma_j^2}\right)}{\sum_{k\neq j}\exp\left(-\frac{D_{kj}^2}{2\sigma_j^2}\right)} \tag{3}$$

   where the perplexity constraint ψ is a tunable hyperparameter which controls the point-specific standard deviation σ according to the following equation.

   $$\psi = 2^{H(P_j)} = 2^{-\sum_{k\neq j} P_{k|j}\log_2(P_{k|j})} \tag{4}$$

   We showed that using a precomputed distance matrix, the tSNE results are essentially the same as passing in the original dataset with the corresponding distance metric and the same initialization.

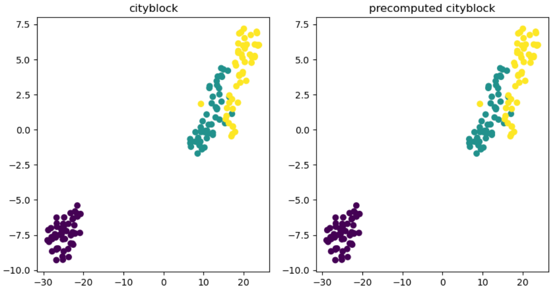

(a) L1 metric

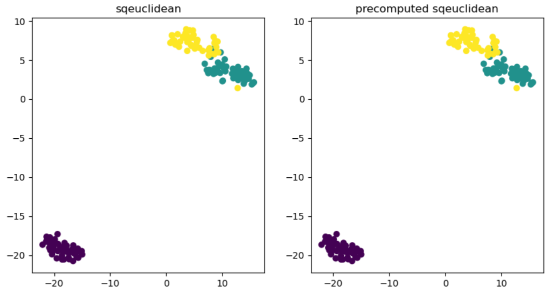

(b) L2 metric

We can confirm that σ was indeed estimated directly for each point, using the perplexity value we chose for the algorithm. In this case it does not matter whether we pass the distance into the algorithm before or after the DoD transformation. Thus, rescaling of σ itself was not enough to solve the scattering problem. We also showed later in our simulations that changing perplexity value could not identify scattering noise as a separable cluster.

2. **The problem with tSNE and UMAP is that there are many tricks for making them work better, and even when you do all the tricks right, there are many aspects of the embedding that seem arbitrary. Some computational researchers reject this outright, claiming that tSNE and UMAP are bad representations of the data and are just there to make "pretty pictures". I wouldn't go as far, I think there's some inherent use for these visualizations, and there are some consistent ways to get good embeddings (see The Art of tSNE). If the authors can compare their tricks to these more standard and consistent tSNE methods, then I think the paper can be a useful contribution to our bag-of-tricks for tSNE and UMAP.**

   In particular, the authors have to show that the crowding problem persists when the tSNE is run in this more standard way: reduce dimensions by PCA, initialize tSNE with PCA, use a perplexity of approx N/100 where N is the number of points. They should also show that the problem persists with different tSNE parameters, such as higher or lower perplexity. Also, UMAP has its own set of parameters that needs to be explored, and all the parameter settings have to be specified in the methods.

   Even though PCA is a frequently used technique to improve the low-dimensional embeddings. There are situations that the scattering noise problem cannot be solved by either PCA preprocessing or PCA initialization. We illustrate this point in the supporting information section:

   PCA as a dimensionality reduction method is often used in combination with t-SNE, mainly to improve computational efficiency by reducing the dimensionality the data set before running t-SNE [1]. There are two ways to incorporate PCA into the t-SNE analysis. First of all, PCA can be used to initialize t-SNE embeddings. For example, we can use the first two principal components as the initialized 2D representations of the dataset, and then t-SNE can carry out gradient descent steps from such initialized coordinates. Secondly, PCA can be used to reduce the dimensionality of the original dataset with PCA as a preprocessing step. This is a standard technique to make the computation more

efficient [1]. By keeping only the first few principal components, we could capture most of the variability in our data with reduced dimensionality.

In our experiments, we found that both PCA initialization and PCA preprocessing did not resolve the scattering noise problem (Supplementary Figure S3). Furthermore, PCA can only be applied to datasets, but not directly to distance matrices. In situations where dissimilarity was calculated not simply as Manhattan / Euclidean distance (e.g. distances among correlation matrices, or optimal transport distances over spiking patterns [13].), it is no longer feasible to apply PCA as a preprocessing step. Overall, the DoD transformation is another useful technique that can be used together with other techniques like PCA. In fact both methods can be combined with their own advantages.

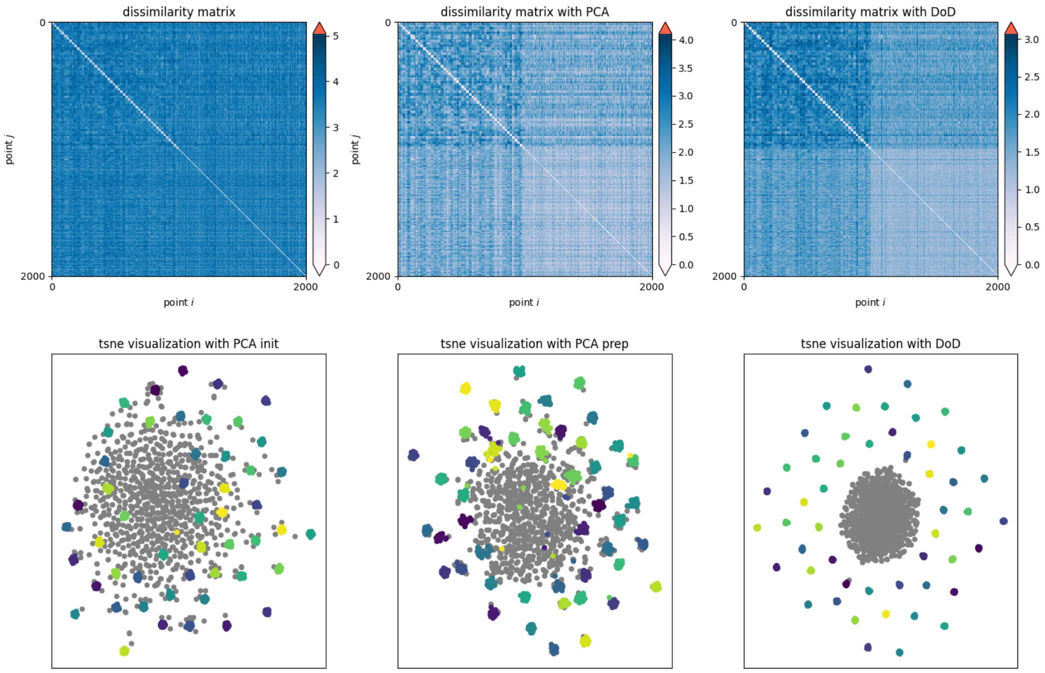

Figure S3: PCA is limited in terms of solving scattering noise problem. 50 clusters, each with 20 points; 1000 scattering noise points; dimensionality of 50; PCA preprocessing uses the first 10 principal components; DoD transformation uses neighborhood size of 10.

By carefully tuning of perplexity value based on the number of points in each cluster and the number of clusters, we did not observe the alleviation of "scattering noise problem". It is important to choose perplexity value, because we do not want to introduce illusional geometry to clusters. However, the choice of perplexity could not help solving the problem of scattering noise problem. We added more discussion on the choice of perplexity parameter in the paper:

Perplexity is an important tunable parameter for the t-SNE algorithm. In the original t-SNE paper, the author suggested that the choice of perplexity value within a reasonable range between 5 and 50 did not seem to influence the experiment results [9]. However, in practice, it has been suggested to use 1% of the total sample size of the dataset [1]. Different choices of perplexity change the behavior of t-SNE [12]. To test whether the scattering noise problem can be solved by t-SNE algorithm itself, we carefully tuned the perplexity value based on the number of points in each cluster and the number of clusters. However, we did not observe the alleviation of the "scattering noise problem". With a higher perplexity value, some local geometrical structures may emerge in the Gaussian clusters as they became more ellipsoidal and also in the random noise cloud. However, we observed that there existed no perplexity value that could make noise points attracted to each other, and the scattering noise problem remained (Supplementary Figure S5).

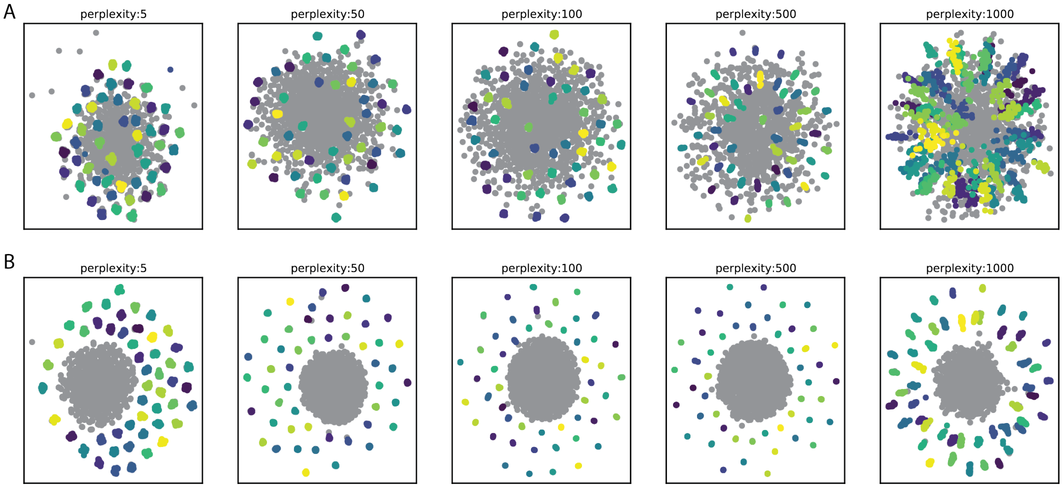

Figure S5: Larger perplexity of t-SNE algorithm does not solve scattering noise problem. 50 clusters, each with 20 points; 1000 noise points; dimensionality of 50; DoD transformation with a neighborhood size of 5; perplexity values are 5, 50, 100, 500, 1000 from left to right. **A.** t-SNE on original distance matrix. **B.** t-SNE on distance matrix after DoD transformation.

3.2. MINOR COMMENTS

1. **heatmaps in all images should be scaled in a more reasonable way (except 4c which is fine) . Right now they just all look red. Perhaps scale between 1% and 99% saturation?**

   We changed all the colormaps to make the distance matrices easier to follow.

2. **the analysis in 2.5 is interesting, but you should mention that the orthogonality of stimulus and spontaneous activity was shown in Stringer et al, Science 2019 for rodents. The activity patterns literally live in different linear subspaces.**

   We included more discussions on the neural manifold of stimulus-evoked response and spontaneous activities including the work of Stringer et al, Science 2019 [14].

3. **Also with respect to 2.5, a comment should be made about the lack of separation of patterns to opposite directions of motion. We know there is direction selectivity in V1, but embedding algorithms have to throw some information away, and the direction selectivity seems to indeed disappear.**

   As the reviewer mentioned, it is very curious why the direction tuning cells are missing from the mouse primary visual cortex. But we confirmed in the Allen Institute Neuropixel datasets that most neurons recorded in this particular session were indeed orientation selective rather than direction selective. Such observation can be confirmed by the bimodal tuning curves of most recorded unit from this particular session. This discussion is also added to the supplementary information:

   Data were downloaded in NWB format from the Allen Neuropixel Brain Observatory. There are in total 32 adult mice in brain observatory 1.1 paradigm with an average age of 112 days. Among them 27 of are male and 5 are female. 16 of them are wild-type mice, and the rest of them are transgenic lines (N = 6, Sst-IRES-Cre/wt; N = 5, Pvalb-IRES-Cre/wt; N = 5, Vip-IRES-Cre/wt). Rodents were head fixed, and presented with different types of visual stimulus in a certain order. In total, six high-throughput Neuropixel probes were inserted into mouse brain and recorded simultaneously from both visual cortical areas and subcortical areas including hippocampus and thalamus. For visual neurons, we include only the units with signal-to-noise ratio larger than 0.5. We estimated the direction selectivity of a single

neuron by its average response to drifting grating stimulus. By investigating the tuning curves of the recorded visual neurons, we found that the majority of them had two peaks with a interval of 180 degrees. The bimodality of the tuning curve suggested that most neurons are orientation selective instead of direction selective (Supplementary Figure S8B).

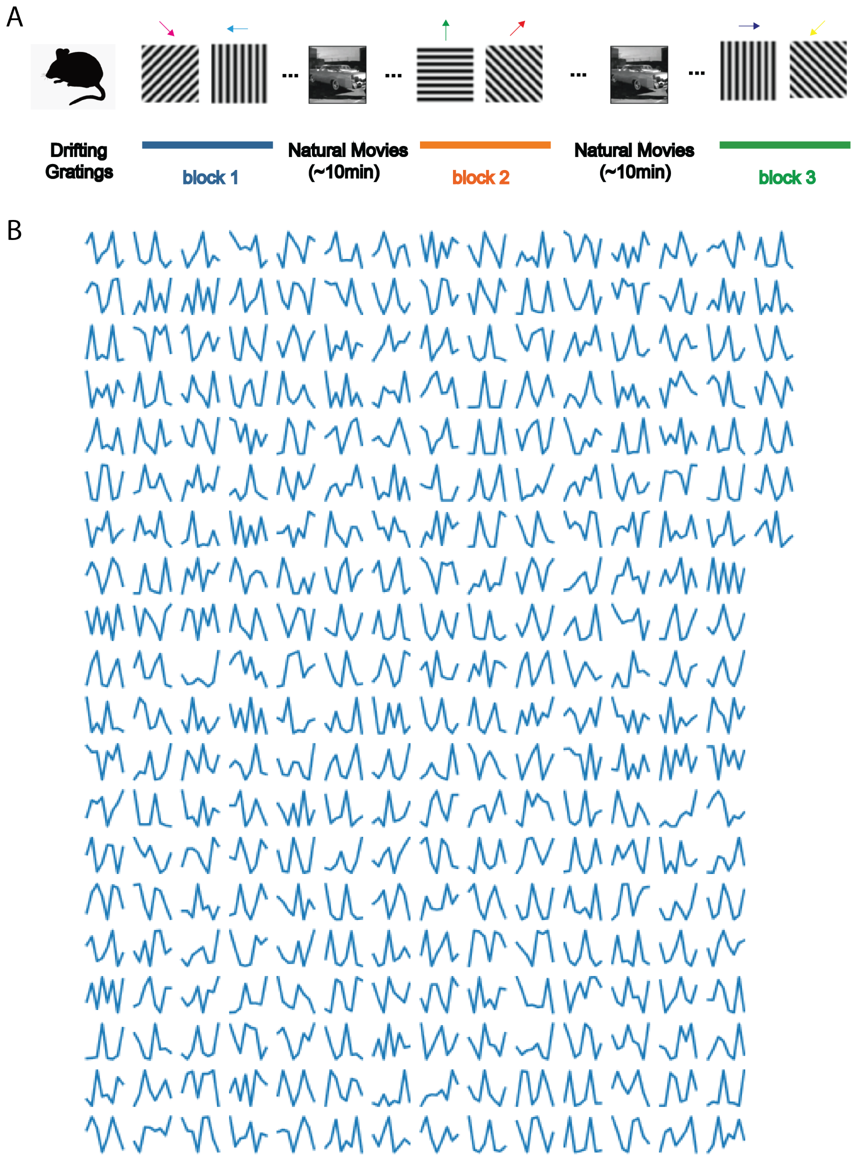

Figure S8: Neural spiking data from Allen Institute. A. Illustration of recording session of drifting grating visual stimulus. **B.** Direction tuning curves of recorded units in session 754829445.

4. **You should add the information about dimensions and number of points to the figure so it's clear without carefully reading the legend. Or at least separate the first two columns from the last two columns so the reader doesn't think it's a progression of 4 settings for one parameter like I originally thought.**

   We added more illustrations in Figure 2 to indicate both the number of cluster/noise points and dimensionality.

5. **Line 228: this is just not true. Choosing the perplexity is one of the most important decision when running t-SNE, as well as a few other things like initialization with PCA and how many PCs to keep from the data (see The Art of TSNE).**

We agreed that choosing perplexity and other parameters when running t-SNE is very important. But we showed that the problem of scattering noise points masking the cluster points can not be solved by tuning perplexity value (Supplementary Figure S5). This line, together with the textbook description of t-SNE algorithm, have now been removed to keep the manuscript more concise.

References

[1] D. Kobak and P. Berens, "The art of using t-sne for single-cell transcriptomics," *Nature communications*, vol. 10, no. 1, pp. 1–14, 2019.

[2] C. Stringer, M. Pachitariu, N. Steinmetz, M. Carandini, and K. D. Harris, "High-dimensional geometry of population responses in visual cortex," *Nature*, p. 1, 2019.

[3] E.-a. D. Amir, K. L. Davis, M. D. Tadmor, E. F. Simonds, J. H. Levine, S. C. Bendall, D. K. Shenfeld, S. Krishnaswamy, G. P. Nolan, and D. Pe'er, "visne enables visualization of high dimensional single-cell data and reveals phenotypic heterogeneity of leukemia," *Nature biotechnology*, vol. 31, no. 6, pp. 545–552, 2013.

[4] W. Li, J. E. Cerise, Y. Yang, and H. Han, "Application of t-sne to human genetic data," *Journal of bioinformatics and computational biology*, vol. 15, no. 04, p. 1750017, 2017.

[5] E. Becht, L. McInnes, J. Healy, C.-A. Dutertre, I. W. Kwok, L. G. Ng, F. Ginhoux, and E. W. Newell, "Dimensionality reduction for visualizing single-cell data using umap," *Nature biotechnology*, vol. 37, no. 1, pp. 38–44, 2019.

[6] R. J. Campello, D. Moulavi, and J. Sander, "Density-based clustering based on hierarchical density estimates," in *Pacific-Asia conference on knowledge discovery and data mining*, pp. 160–172, Springer, 2013.

[7] J. A. Gallego, M. G. Perich, L. E. Miller, and S. A. Solla, "Neural manifolds for the control of movement," *Neuron*, vol. 94, no. 5, pp. 978–984, 2017.

[8] J. P. Cunningham and M. Y. Byron, "Dimensionality reduction for large-scale neural recordings," *Nature neuroscience*, vol. 17, no. 11, pp. 1500–1509, 2014.

[9] L. v. d. Maaten and G. Hinton, "Visualizing data using t-sne," *Journal of machine learning research*, vol. 9, no. Nov, pp. 2579–2605, 2008.

[10] G. E. Hinton and S. T. Roweis, "Stochastic neighbor embedding," in *Advances in neural information processing systems*, pp. 857–864, 2003.

[11] L. McInnes, J. Healy, and J. Melville, "Umap: Uniform manifold approximation and projection for dimension reduction," *arXiv preprint arXiv:1802.03426*, 2018.

[12] M. Wattenberg, F. Viégas, and I. Johnson, "How to use t-sne effectively," *Distill*, vol. 1, no. 10, p. e2, 2016.

[13] L. Grossberger, F. P. Battaglia, and M. Vinck, "Unsupervised clustering of temporal patterns in high-dimensional neuronal ensembles using a novel dissimilarity measure," *PLoS computational biology*, vol. 14, no. 7, p. e1006283, 2018.

[14] C. Stringer, M. Pachitariu, N. Steinmetz, C. B. Reddy, M. Carandini, and K. D. Harris, "Spontaneous behaviors drive multidimensional, brainwide activity," *Science*, vol. 364, no. 6437, p. eaav7893, 2019.
