# Supplementary material for: Improved visualization of high-dimensional data using the distance-of-distance transformation
Source: PLoS Comput Biol. 2022 Dec 20;18(12):e1010764. doi: 10.1371/journal.pcbi.1010764 (PMC9812310; doi:10.1371/journal.pcbi.1010764)
Supplement: Attachment — Submitted filename: response_to_reviewers.pdf [file pcbi.1010764.s028.pdf]

Response to reviewers for: Improved visualization of high-dimensional data using the distance-of-distance transformation

Jinke Liu[1,2] and Martin Vinck[1,2]

[1]Ernst Strüngmann Institute (ESI) for Neuroscience in Cooperation with Max Planck Society, Frankfurt, Germany
[2]Donders Centre for Neuroscience, Department of Neuroinformatics, Radboud University Nijmegen, Nijmegen, Netherlands

October 20, 2022

Contents

1. Review 1 **2**

2. Review 2 **7**

3. Review 3 **8**

1. Review 1

The revision is a MASSIVE improvement, with all raised points adequately addressed. I wish I saw such a thorough and comprehensive revision more often! I only have minor comments left.

We thank the reviewer for their positive comments. We have addressed the remaining minor comments in the revised manuscript accordingly.

- **Fig S1A – to illustrate the point better, you could remove K=10 and K=15 from the top row (K=5 and K=20 are nearly identical anyway) and rather add K=50 and K=100, which would show how the performance degrades for larger K.**

  We thank the reviewer for the suggestion. Now K ranges from 10 to 100 in the first simulation (See Figure S1A). With K increasing, the performance of DoD transformation stays relatively robust. Even though the performance degradation is less severe than in Figure S1B when there are more clusters, there is some distortion of the geometry with a larger K. The textual description of the supplementary figure has also been updated.

- **Fig S1A – replace ”original” $\longrightarrow$ ”original embedding”? The word ”original” suggests to me that this panel shows the original 2D data which is of course not the case as the generated data are higher-dimensional**

  We have followed the recommendation of the reviewer and replaced “original” by “original embedding”.

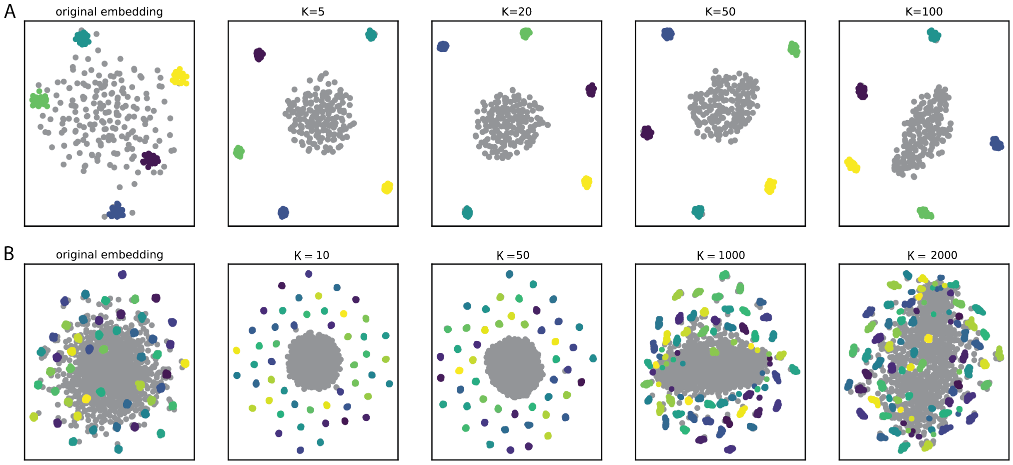

Figure S1: Effect of neighborhood size K. A. 5 clusters, each with 20 points; 200 scattering noise points; dimensionality of 50; Original embedding (left) and DoD transformation with a neighborhood size ranging from 5 to 100. **B.** 50 clusters, each with 20 points; 1000 scattering noise points; dimensionality of 50; Original embedding (left) and DoD transformation with a neighborhood size of 10, 50, 1000 and 2000.

- **Fig S6 – ”K” instead of ”number of neighbors”? That’s how it was in Fig S1**

  Following the reviewer’s suggestion, we have replaced “number of neighbors” with “K”.

- **Fig S6 – corr coefficients can be rounded e.g. to two or three digits, currently the precision looks odd**

  We followed the reviewer’s recommendation and rounded the correlation coefficients to two digits and updated the figure (See Figure S6).

- **Fig S7 – here I am confused: are the simulated data 2D or higher-dimensional? If the data are higher-dimensional, then I don’t understand why the ”original” t-SNE shows smaller density for the lower-left cluster. t-SNE adjusts the kernel width to reach a given perplexity and typically does not preserve the cluster density.**

The simulated data are indeed high-dimensional ($D = 50$). This is mentioned in the caption. As the reviewer pointed out, whether the low-dimensional t-SNE embedding reflects the density depends on the choice of perplexity. In our simulations, perplexity is set to a default value of 30. One could choose a smaller perplexity such as 10 or 15 to make the clusters look as if they are similarly dense (See the Figure for the Reviewer below).

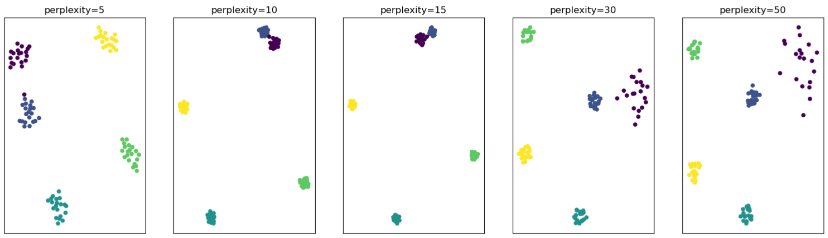

However, for consistency we prefer to present the simulations with the original value of 30. This does not harm the gist of the figure showing that DoD transformation does not introduce distortions in the absence of scattering noise points. But to avoid confusion, we have added a note to the caption that one could lower the perplexity to make the densities across clusters look more similar in the original embedding. And Figure S7 is now combined with Figure S6 in our revised manuscript.

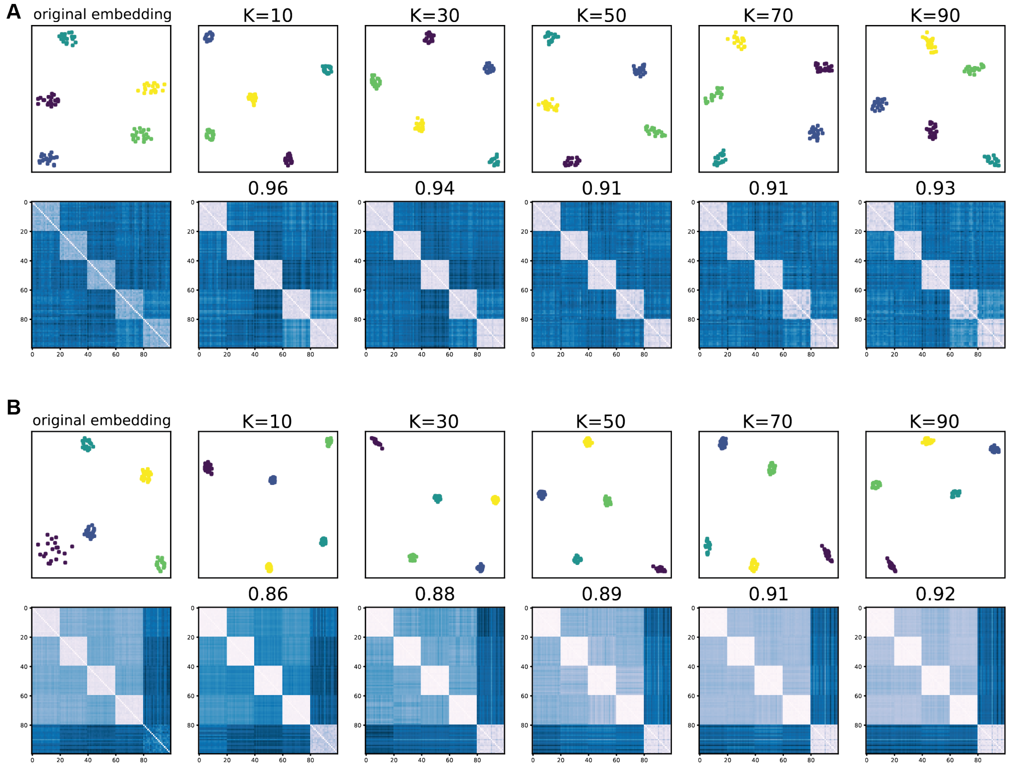

Figure S6: DoD transformation does not distort the clustering. **A.** 5 clusters, each with 20 points; dimensionality of 50; DoD transformation with neighborhood size ranging from 5 to 30. **B.** 5 clusters, each with 20 points; dimensionality of 50; DoD transformation with neighborhood size ranging from 5 to 30. All clusters were generated from multivariate Gaussian distribution and one of them is with a larger standard deviation (0.5) than the rest (0.2). Note that the perplexity of t-SNE could influence the visualization of the original embedding. Here in all the simulations, perplexity is set to a default value of 30. A smaller perplexity value such as 10 could visualize the low-density cluster equally dense as the other clusters.

- **"introduces very limited distortions and preserve" ⟶ "... preserves"**

  This grammar mistake has been fixed.

- **Fig S11 – red dots in the matrix look really weird and confusing. If it's simpler all values above the top colorbar limit, then I would suggest to color them dark blue (same shade as in the top end of the colorbar).**

  Indeed, values above the upper limit of the colorbar were colored in red, because we do not want the outliers to mask the patterns in the heatmap. We now change the color from red to dark blue in Figure S11 to cause less confusion.

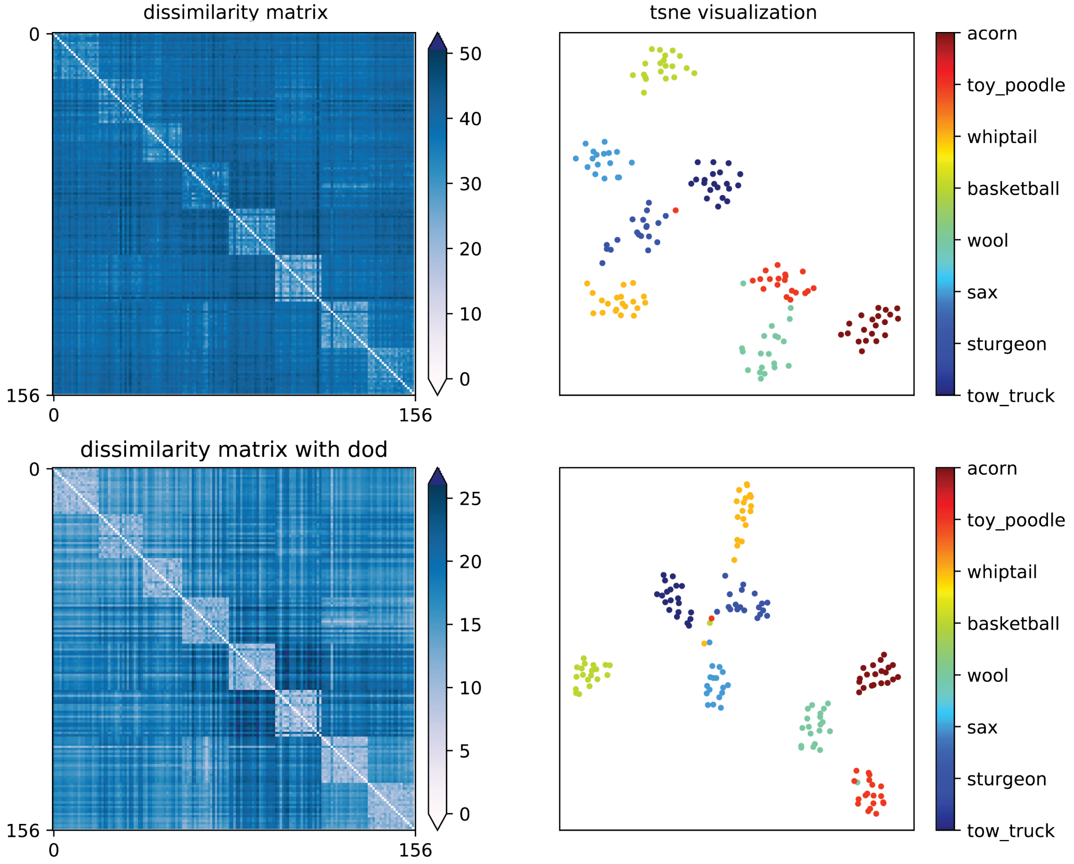

Figure S11: Object manifolds were not distorted by the DoD transformation. 2D t-SNE embeddings for 8 random ImageNet classes maintain clustering patterns after the DoD transformation.

- **”In situations where there are less noise than the number of clusters” – cumbersome formulation. ”... where the number of noise points is smaller than the number of non-noise points”?**

  We thank the reviewer for the suggestion, the corresponding sentence in the supplementary information has been replaced.

  In situations where the number of noise points is smaller than the number of non-noise points, DoD transformation still succeeded in putting all noise points together as a separate cluster.

- **Fig S2 – what was the K here? Same as for DoD computation?**

  In this simulation, the parameter K used for DoD transformation is 5. It is essentially the same experimental setting as in Figure 1 but without any class labels. This is now mentioned in S2 Text.

- **”(e.g. distances among correlation matrices, or optimal transport distances over spiking patterns [13].)” – remove the period**

  We removed the period, the change can be found in the revised supplementary information.

- **line 69: unclear why you use L1 distance here and not L2. Same about line 72. Would it work with L2 distance in either the first, or the second, or both places? Could be nice to show that empirically e.g. for the situation in Figure 1**

  DoD transformation works in both cases regardless of which distance metric we use to construct the original distance matrix. We have added a sentence on line 147 of the manuscript that an almost identical result can be obtained with L2 norm:

  The DoD transformation helps to separate the noise cloud from the clusters regardless of which distance metric we use to construct the original distance matrix $\mathcal{D}$. The results obtained by using L2 metric are almost identical to those by using L1 metric.

  As for the equation 1 of DoD transformation, we change the notation $||.||_1$ to $|.|$ to avoid confusion, because $d_{n,i}$ and $d_{n,j}$ are both scalars.

  Mathematically speaking, given a dataset $X \in \mathbb{R}^{N\times D}$ with N samples and D features, the distance matrix is constructed as $\mathcal{D} \in \mathbb{R}^{N\times N}$ with either L1 or L2 distance metric, where $d_{i,j} = ||X_i - X_j||_1$ or $||X_i - X_j||_2$. For any two data points $X_i, X_j \in \mathbb{R}^D$, we found their sets of K nearest neighbors I and J respectively. Then we manipulated the original distance matrix $\mathcal{D}$ to yield another new distance matrix $\mathcal{F}$. We take the average L1 difference between the two points' distances to the selected neighborhood as the new distance as following,

  $$f_{i,j} \equiv \frac{1}{2K} \sum_{n \in I \cup J} |d_{n,i} - d_{n,j}|$$

- **Fig 4C – it is confusing to me that N grows from top to bottom and not from bottom to top...**

  We rearranged Figure 4C, so that N grows from bottom to top.

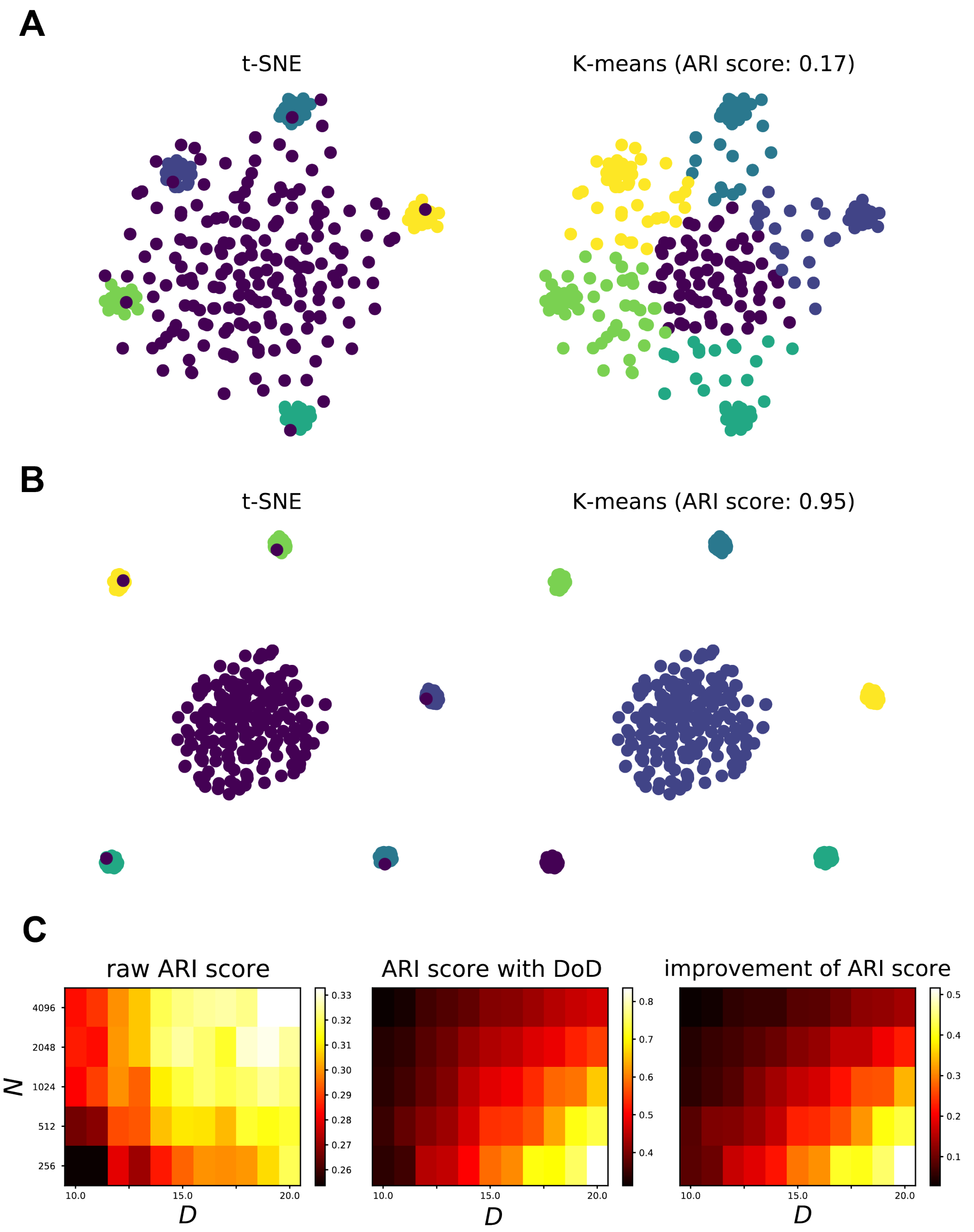

- **line 222 – please state here what the dimensionality is (4096?)**

  The dimensionality of the feature vector is mentioned in the method section. Here from line 331 to 334 of the revised manuscript, we restate the dimensionality to make it clearer.

  By transformations of input data through multiple convolutional layers, VGG16 represents an image in the fully connected layer as a high-dimensional feature vector of length 4096.

2. Review 2

The authors have addressed the points from my previous review adequately. The article is well-presented and interesting, however it will need to be proofread for spelling and grammar errors.

1. **Inconsistencies in writing ”neighbour” versus ”neighbor”, ”DoD” versus ”Distance-of-Distance”, etc**
2. **Author summary: typo with ”hihg-dimensional”. I believe it should also be ”low-dimensional” instead of ”low dimensional” to be consistent**
3. **The paragraph at 173 is unclear and needs to be rewritten. It has several grammar and spelling errors: ”less noise points than...” instead of ”fewer noise points than...”, ”our method are” instead of ”our method is”, ”one cluster has low density than” instead of ”one cluster has lower density than”**
4. **381: should be ”requires computation of the...”**
5. **384: should be ”an NxN...”**
6. **393: I believe the authors mean to convey that their method provided improved or more accurate conclusions rather than simply ”qualitatively different” conclusions (at least that’s how I see it!)**

We thank the reviewer for carefully pointing out all the grammar and spelling errors. With further meticulous proofreading, all such mistakes have been fixed.

3. Review 3

The authors have addressed all my comments in a satisfactory manner. Thank you.

We thank the reviewer for his/her comments.
