# Supplementary material for: Improved visualization of high-dimensional data using the distance-of-distance transformation
Source: PLoS Comput Biol. 2022 Dec 20;18(12):e1010764. doi: 10.1371/journal.pcbi.1010764 (PMC9812310; doi:10.1371/journal.pcbi.1010764)
Supplement: Attachment — Submitted filename: letter_to_reviewer.pdf [file pcbi.1010764.s029.pdf]

Response to reviewers for: Improved visualization of high-dimensional data using the distance-of-distance transformation

Jinke Liu[1,2] and Martin Vinck[1,2]

[1]Ernst Strüngmann Institute (ESI) for Neuroscience in Cooperation with Max Planck Society, Frankfurt, Germany
[2]Donders Centre for Neuroscience, Department of Neuroinformatics, Radboud University Nijmegen, Nijmegen, Netherlands

November 15, 2022

Contents

1. Review 1 **2**

1. Review 1

In Formula 1, I don't understand why the fraction before the sum is $\frac{1}{2K}$, as the size of the $I \cup J$ union may be smaller than $2K$. Should $2K$ in that formula be replaced by $|I \cup J|$?

We thank the reviewer for carefully pointing out this mistake. If point i and j shares a point n in their neighborhoods, i.e. $I \cap J \neq \emptyset$, we consider n twice in our computation. We corrected the equation in our manuscript as follows:

$$f_{i,j} \equiv \frac{1}{2K} \left(\sum_{n \in I} |d_{n,i} - d_{n,j}| + \sum_{m \in J} |d_{m,i} - d_{m,j}| \right) \tag{1}$$
