# Supplementary material for: Improved visualization of high-dimensional data using the distance-of-distance transformation
Source: PLoS Comput Biol. 2022 Dec 20;18(12):e1010764. doi: 10.1371/journal.pcbi.1010764 (PMC9812310; doi:10.1371/journal.pcbi.1010764)
Supplement: Attachment — Submitted filename: Review.docx [file pcbi.1010764.s026.docx]

**Review: Noise-robust low-dimensional embedding using distance-of-distance transformation**

Overall

This is a manuscript on improving dimensionality reduction through an intermediate transformation of data that filters noise by essentially condensing noise points so that they do not clutter data that represent signal. It is an interesting and relatively straightforward improvement that will be applicable to data that exist in high dimensions with a moderate number of points (the authors state that its improvements slow down with larger N).

The authors do a good job of constructing the idea from first principles, creating an appropriate simulation, and finally applying the method to real data. The authors have not provided a link to the code and data repositories, though I assume that they are awaiting review before making their code public.

Comments

- Please provide a link to code/data repositories
- The UMAP minimum_distance parameter (particularly low minimum distance) and the t-SNE parameter perplexity can address the tightness of clusters in low dimensions. This can help separate clusters. It would be worth it to run the data from Figure 4A again with a higher perplexity as a sanity check (I suspect your transformation will still be better)
- “Drifting grating” is not defined – for those unfamiliar with neuronal stimulus research a short description (e.g. “moving bars”) would be helpful.
- The labels for scale bars on most heat maps are confusing because of spacing between the plots and scales. Figure 4C is clear, however, and is a good template for the other figures.
- For Figure 6, the spontaneous activities in gray are exemplified by the raster plot in 6B, correct?
- In your discussion you mention that the algorithm is computationally intensive since it calculates an NxN distance matrix. Providing runtimes for some of the transformations that you carried out would be very useful.
- Is there a point at which you would consider this transformation’s tradeoffs to be too onerous? I’m referring specifically to Figure 3C/D, where large N reduces the effectiveness of this transformation, as well as your comments on the computational intensity of transformation an NxN matrix in D dimensions.
